# A Hsp40 Chaperone Protein Interacts with and Modulates the Cellular Distribution of the Primase Protein of Human Cytomegalovirus

**DOI:** 10.1371/journal.ppat.1002968

**Published:** 2012-11-01

**Authors:** Yonggang Pei, Wenmin Fu, Ed Yang, Ao Shen, Yuan-Chuan Chen, Hao Gong, Jun Chen, Jun Huang, Gengfu Xiao, Fenyong Liu

**Affiliations:** 1 State Key Laboratory of Virology, College of Life Sciences, Wuhan University, Wuhan, Hubei, China; 2 Division of Infectious Diseases and Vaccinology, School of Public Health, University of California, Berkeley, California, United States of America; University of Alabama at Birmingham, United States of America

## Abstract

Genomic DNA replication is a universal and essential process for all herpesvirus including human cytomegalovirus (HCMV). HCMV UL70 protein, which is believed to encode the primase activity of the viral DNA replication machinery and is highly conserved among herpesviruses, needs to be localized in the nucleus, the site of viral DNA synthesis. No host factors that facilitate the nuclear import of UL70 have been reported. In this study, we provided the first direct evidence that UL70 specifically interacts with a highly conserved and ubiquitously expressed member of the heat shock protein Hsp40/DNAJ family, DNAJB6, which is expressed as two isoforms, a and b, as a result of alternative splicing. The interaction of UL70 with a common region of DNAJB6a and b was identified by both a two hybrid screen in yeast and coimmunoprecipitation in human cells. In transfected cells, UL70 was primarily co-localized with DNAJB6a in the nuclei and with DNAJB6b in the cytoplasm, respectively. The nuclear import of UL70 was increased in cells in which DNAJB6a was up-regulated or DNAJB6b was down-regulated, and was reduced in cells in which DNAJB6a was down-regulated or DNAJB6b was up-regulated. Furthermore, the level of viral DNA synthesis and progeny production was increased in cells in which DNAJB6a was up-regulated or DNAJB6b was down-regulated, and was reduced in cells in which DNAJB6a was down-regulated or DNAJB6b was up-regulated. Thus, DNAJB6a and b appear to enhance the nuclear import and cytoplasmic accumulation of UL70, respectively. Our results also suggest that the relative expression levels of DNAJB6 isoforms may play a key role in regulating the cellular localization of UL70, leading to modulation of HCMV DNA synthesis and lytic infection.

## Author Summary

Genomic DNA replication is highly conserved across all herpesviruses including human cytomegalovirus (HCMV) and is the target for most of the current FDA-approved anti-herpes therapeutic agents. Little is known about how UL70, which is believed to encode the primase activity of the viral DNA replication machinery and is essential for genomic replication, is imported to the nuclei, the site of viral DNA synthesis. In this study, we demonstrated that the HCMV primase interacts with a highly conserved and ubiquitously expressed chaperone protein DNAJB6 that belongs to the heat shock protein 40 (Hsp40) family. As a result of alternative splicing, DNAJB6 is expressed as two isoforms, a and b. While DNAJB6b promotes cytoplasmic accumulation of the viral primase, DNAJB6a enhances its nuclear distribution, representing the first example of a cellular factor involved in facilitating nuclear import of a herpesvirus primase. Our study suggests that the relative expression level of DNAJB6 isoforms may represent a novel mechanism for modulating HCMV lytic replication by regulating the cellular localization of the viral primase. Furthermore, our results raise the possibility of developing new strategies for treating herpesvirus replication by modulating the cellular distribution of the primase with altered expression of a cellular protein.

## Introduction

Human cytomegalovirus (HCMV), is a member of the herpesvirus family, which includes herpes simplex virus 1 (HSV-1), Epstein Barr virus (EBV), and Kaposi's sarcoma-associated herpesvirus (KSHV) [1]. This virus is the leading viral cause of congenital abnormalities and mental retardation in newborns, and causes significant morbidity and mortality in immunocompromised individuals such as AIDS patients and organ transplant recipients. The hallmarks of HCMV pathogenesis include infection of a wide range of tissues and cells such as neuronal cells, and the establishment of lytic and latent infection in many of these tissues [1], [2]. During lytic infection, the viral DNA genome is replicated in the nucleus of infected cells [3]. The process of genomic DNA replication is highly conserved across all herpesviruses and is the target for most of the current FDA-approved anti-herpes therapeutic agents [4]. Understanding the mechanism of herpesviral genomic DNA replication is important for the development of new strategies to treat infections of these viruses including HCMV.

As in the genomic DNA replication of other hepersviruses, a conserved set of six proteins are believed to provide the core functions for HCMV DNA replication: the DNA polymerase (UL54), the associated polymerase processivity factor (UL44), the single-stranded DNA binding protein (UL57), the helicase (UL105), the primase (UL70), and the primase-associated factor (UL102) [1], [3], [5], [6]. Primase is a DNA-dependent RNA polymerase that synthesizes a short RNA primer for the Okazaki fragments made by DNA polymerase during discontinuous DNA replication [7], [8]. Herpesviruses usually form a helicase-primase complex, which consists of UL105, UL70 and UL102 in HCMV [9], [10], [11], [12]. This heterotrimeric complex possesses three main activities: primase, DNA-dependent NTPase, and 5′-3′ helicase. The helicase tracks along the lagging strand and unwinds the DNA in front of the replication fork, and the NTPase provides the energy needed for unwinding [1], [3]. Although the precise role of each subunit needs further investigation, it would be expected, by analogy with observations in other herpesviruses (e.g. HSV-1), that an assembled subcomplex containing UL105 and UL70 subunits retains all three activities, while the UL102 subunit modulates these activities [9], [13], [14], [15].

Studies have been carried out to determine the site of HCMV DNA replication and to investigate the functions of each of the core proteins and their interactions in viral DNA replication [1]. During lytic infection, viral DNA replication compartments, which are the sites for both input viral genome deposition and gene transcription, are assembled into dot-like subnuclear structures located in or close to specific nuclear regions called promyelocytic leukemia protein (PML)-associated nuclear bodies (also known as PML oncogenic domains [PODs]) or nuclear domain 10 (ND10) [16], [17], [18], [19]. However, little is known about the pathway by which the HCMV replication machinery is assembled. It has been shown that HCMV DNA replication proteins, including UL44, UL54, and UL57, which possess nuclear localization signal sequences (NLS), interact with human cellular importin to facilitate the newly synthesized viral proteins to be imported into the nucleus through the nuclear pore complex [20], [21], [22], [23], [24], [25]. However, no NLS has been reported in UL70 and its homologous proteins in other herpesviruses. Little is known about how UL70 is imported in the nuclear compartment where viral DNA replication occurs [26]. It has not been reported whether any host proteins facilitate the nuclear import of UL70 or other herpesvirus primases by interacting with these proteins.

Cellular heat shock proteins (Hsps) are a family of molecular chaperones that contribute to prevent aggregation and participate in the refolding of misfolded proteins during environmental stresses such as heat shock, UV irradiation, and microbial infections [27], [28]. The Hsp40/DnaJ family of chaperone proteins has a highly conserved amino acid sequence called the “J-domain” and achieves its biological functions by interacting with diverse cellular proteins [29]. Typically, the Hsp40/DnaJ proteins play a critical role through binding with the cochaperone Hsp70 to stimulate ATP hydrolysis and are involved in the regulation of important cellular processes such as protein degradation, exocytosis, and endocytosis [27], [28]. It has been shown that several Hsps are important for the replication of viruses [29], [30], [31], [32], [33]. For example, Hsp90 has been shown to be required for the nuclear import of the HSV-1 polymerase [30], [34] while Hsp90β and DNAJB11 interact with KSHV K1 protein and are required for its anti-apoptotic activity [35].

In order to gain a better understanding of the HCMV DNA replication process, we in this study carried out a yeast two-hybrid (YTH) screen to identify cellular factors that potentially interact with UL70. Our results provide the first direct evidence that UL70 specifically interacts with human protein DNAJB6, which belongs to the heat shock protein (Hsp) Hsp40/DNAJ family [29], [36], [37]. As a result of alternative splicing, DNAJB6 in humans is expressed as two protein isoforms, DNAJB6a of 326 amino acids (also called as DNAJB6 isoform a or Mrj [L]) and DNAJB6b of 241 amino acids (also called DNAJB6 isoform b or Mrj [S]) [38]. These two proteins share the N-terminal 231 amino acids including the highly conserved DnaJ homology region but differ at the less conserved C-terminal region in which a NLS is present in the longer DNAJB6a, a protein predominantly localized in the nuclei, but is absent in the shorter DNAJB6b, a protein primarily localized in the cytoplasm [38], [39], [40].

Our results showed that the level of UL70 in the nuclei is increased in cells in which DNAJB6a is up-regulated or DNAJB6b is down-regulated, and is reduced in cells in which DNAJB6a is down-regulated or DNAJB6b is up-regulated. Importantly, the levels of viral DNA synthesis and progeny production are similarly correlated with the expression levels of DNAJB6a and b. These results indicate that DNAJB6a and b enhance the nuclear import and cytoplasmic accumulation of UL70, respectively. Our study suggests that DNAJB6 may play a key role in regulating the cellular localization of UL70, leading to modulation of viral DNA synthesis and lytic productive infection.

## Results

### Interaction between UL70 and DNAJB6 identified by yeast two-hybrid analysis

**Figure 1:**
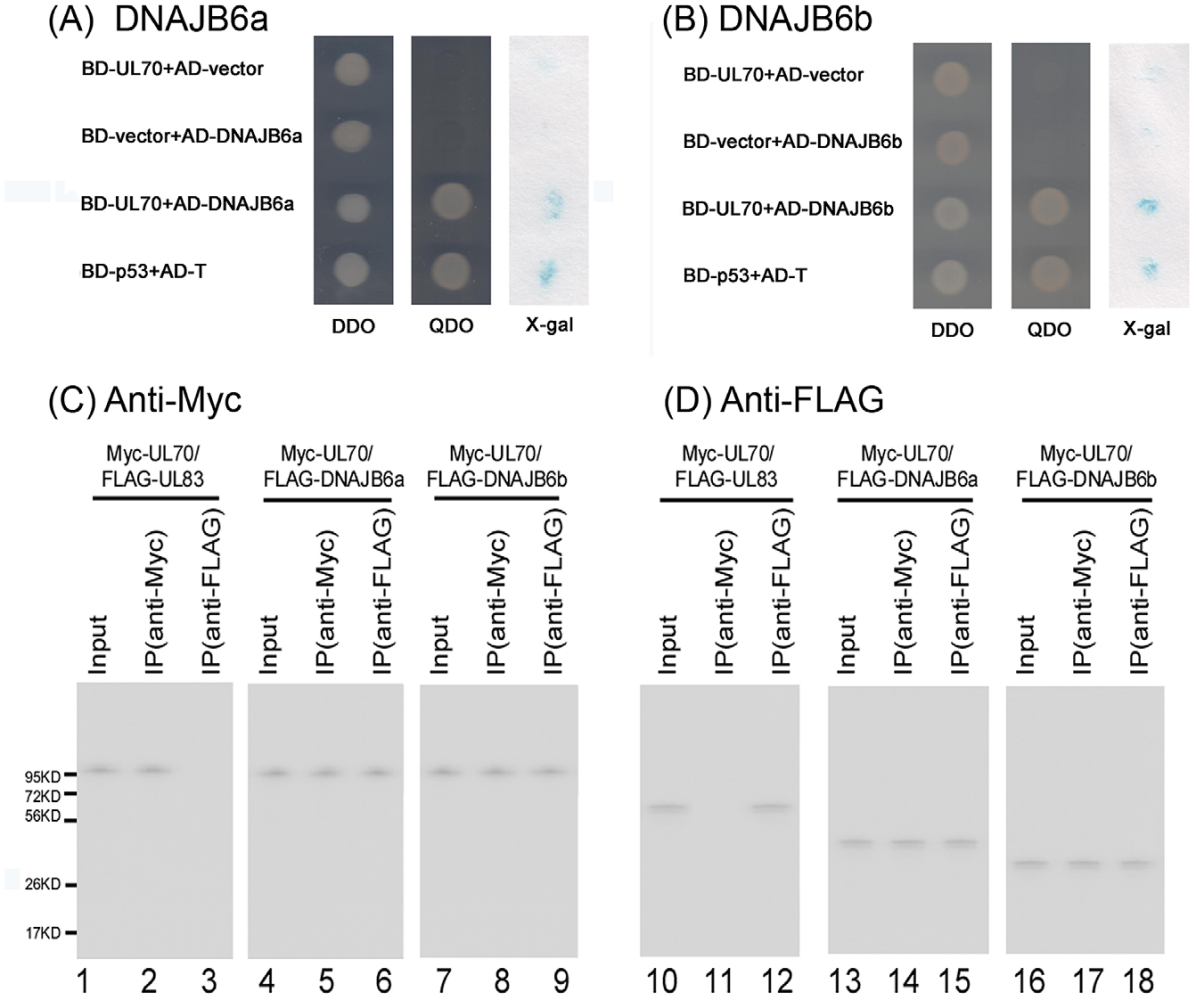
Identification of the interaction between UL70 and two isoforms of DNAJB6 by yeast two-hybrid screen and coimmunoprecipitation. (A–B) Yeast strain AH109 was co-transformed with the combination of one BD and one AD plasmid as indicated (A–B). Transformed yeast cells containing both plasmids were first grown on SD-minus Trp/Leu plates (DDO) to maintain the two plasmids, then colonies were replica plated onto SD-minus Trp/Leu/Ade/His plates (QDO) and also subjected to beta-gal activity test (x-gal) by filter lift staining. No interactions were identified between BD-UL70 and control empty vector AD-vector or between the control empty vector BD-vector and AD-DNAJB6 isoform a and b. Positive interactions were identified between BD-UL70 and AD-DNAJB6 isoform a and b, as well as BD-p53 and AD-T, which served as a positive control. All samples in the three panels (DDO, QDO, and X-gal) were prepared from the same experiments at the same time and in parallel. (C–D) Human U251 cells were co-transfected with a combination of two plasmids expressing FLAG- and Myc-tagged proteins, and then harvested at 48 hours posttransfection. The input protein samples (80 µg) (Input) (lanes 1, 4, 7, 10, 13, and 16) and samples (15 µg) that were either immunoprecipitated with anti-Myc (IP (anti-Myc)) (lanes 2, 5, 8, 11, 14, and 17) or anti-FLAG antibodies (IP (anti-FLAG)) (lanes 3, 6, 9, 12, 15, and 18) were separated on SDS-containing polyacrylamide gels, and assayed with Western blot analysis using anti-Myc (anti-Myc) (C) and anti-FLAG antibodies (anti-FLAG) (D), respectively.

Yeast two-hybrid (YTH) analysis was carried out to identify potential cellular factors that interact with UL70. In these experiments, the DNA sequence encoding the UL70 open reading frame (ORF) was cloned into the pGBKT7 yeast expression vector, and the YTH screen was performed by transformation of *S. cerevisiae* AH109 containing pGBKT7-UL70 with a cDNA library derived from human fetal brain [41]. Those yeast cells that grew on synthetic dropout (SD) medium lacking four nutrients: tryptophan, leucine, adenine, and histidine (SD-minus Trp/Leu/Ade/His, QDO), and yielded blue signals on colony-lift filter test for β-galactosidase activity were identified and defined as primary positive clones (Figure 1A–B). One of the positive constructs, pACT2-DNAJB6b, which contained the coding sequence of DNAJB6 isoform b, was consistently identified to interact with UL70 in our yeast two hybrid screens (Figure 1B). DNAJB6 is a member of the type II group of Hsp40/DNAJ co-chaperone proteins [29], [39], [42]. Two isoforms, DNAJB6a and DNAJB6b, are found in humans, as a result of alternative splicing [38]. In addition to DNAJB6b, the interaction between DNAJB6a and UL70 was also found to be positive in the YTH experiments (Figure 1A). The vectors did not appear to be responsible for the observed interactions as no positive signals were found in the interactions between BD-UL70 and AD-vector or between BD-vector and AD-DNAJB6a or -DNAJB6b (Figure 1A–B). BD-p53 contains the sequence coding for murine p53 protein fused to the binding domain of GAL4 while AD-T contains the sequence coding for the SV40 large T-antigen fused to the activation domain of GAL4. Murine p53 and SV40 T-antigen interacted with each other and served as a positive control (Figure 1A–B).

### Interaction of UL70 and DNAJB6 in human cells identified by coimmunoprecipitation (co-IP)

To further determine whether the interaction between UL70 and DNAJB6a or DNAJB6b occurs in human cells, a co-IP assay was performed in order to validate the results from the YTH system. In these experiments, the sequences coding for UL70 and DNAJB6 (a and b) were cloned into mammalian expression vectors pCMV-Myc and pRK11-FLAG to generate constructs pCMV-Myc-UL70 and pRK11-FLAG-DNAJB6 (a and b), in which each ORF was expressed as a fusion protein with an amino terminal Myc and FLAG epitope tag, respectively. Human astrocytoma U251 cells were transfected with the mammalian expression constructs and were harvested at 48 hours. Protein samples were separated electrophoretically on SDS-containing gels, transferred electrically to membranes, and reacted with anti-FLAG and anti-Myc antibodies. HCMV UL70, DNAJB6a, and DNAJB6b were detected as proteins of about 100, 40, and 30 kD, respectively (Figure 1C–D), consistent with their coding sequences of 946, 326, and 241 amino acids, as predicted from the HCMV Towne_BAC_ and human genomic sequences [38], [43].

Our co-IP experiments showed that UL70 was found to be associated with both DNAJB6a and b in U251 cells transfected with constructs expressing the Myc-tagged UL70 and the FLAG-tagged DNAJB6a (or b) proteins (Figure 1C–D). Protein lysates from the transfected cells were first either immunoprecipitated with anti-Myc or anti-FLAG, and then immunoblotted with antibodies against the Myc and FLAG epitope tags. The Myc-tagged UL70 was co-precipitated with the FLAG-tagged DNAJB6a and b (Figure 1C–D). In contrast, we observed, in control experiments, no significant binding or co-precipitation between the Myc-tagged UL70 and the FLAG-tagged UL83 of HCMV, which is not known to interact with UL70 and serves as a negative control (Figure 1C–D, lanes 1–3 and 10–12) [44]. These results confirmed the specificity of the co-IP assay and suggest that the UL70-DNAJB6 interaction may occur in human cells during HCMV infection. Similar results were also found in HeLa cells, 293T cells, and HCMV-infected U251 cells transfected with the constructs expressing the Myc-tagged UL70 and FLAG-tagged DNAJB6 proteins (data not shown).

As a chaperone protein, DNAJB6 may non-specifically bind to proteins that contain unnatural tagged sequences or are over-expressed. Two sets of experiments were further carried out to determine if UL70 specifically interacts with endogenous DNAJB6 in human cells and in the presence of HCMV infection. In the first set of experiments, U251 cells were transfected with the mammalian expression constructs expressing Myc-tagged UL70 as well as HCMV UL25 protein and then infected with HCMV. UL25 was not known to interact with DNAJB6 and was used as a control to determine if DNAJB6 non-specifically interacts with any Myc-tagged HCMV proteins. Protein lysates from the cells were first either immunoprecipitated with anti-Myc or anti-DNAJB6a or b, and then immunoblotted with antibodies against the Myc epitope tag and DNAJB6a or b. The Myc-tagged UL70 was co-precipitated with the endogenous DNAJB6a (Figure S1, lanes 6 and 11)(Supporting Information). In contrast, we observed, in control experiments, no significant binding or co-precipitation between the Myc-tagged UL25 and DNAJB6a (Figure S1, lanes 1–3 and 7–9). Co-IP experiments using the anti-DNAJB6b antibody also revealed that DNAJB6b co-immunoprecipitated with Myc-tagged UL70 but not UL25 (data not shown). Similar results were also observed in uninfected cells that were transfected with pCMV-Myc-UL70 (data not shown). These results suggest that DNAJB6a and DNAJB6b do not non-specifically interact with the tag sequence but rather specifically interact with UL70 in human cells.

The second set of experiments was to determine if the native (untagged) UL70 protein is associated with DNAJB6 in human cells during HCMV infection. We have expressed UL70 in *E. coli*, and used the expressed protein as the antigen to generate an anti-UL70 monoclonal antibody. To determine if DNAJB6 may interact with other viral DNA replication proteins in addition to UL70, we also investigated if DNAJB6 is associated with UL44, which encodes the HCMV polymerase processivity factor essential for viral DNA replication [1]. Protein lysates from HCMV-infected U251 cells were first either immunoprecipitated with anti-UL70, anti-UL44, or anti-DNAJB6a or b, and then immunoblotted with antibodies against UL70, UL44, and DNAJB6a or b. UL70 was co-precipitated with the endogenous DNAJB6a (Figure S2, lanes 9 and 11)(Supporting Information) while we observed no significant binding or co-precipitation between UL44 and DNAJB6a (Figure S2, lanes 1–6). Co-IP experiments also revealed that DNAJB6b co-immunoprecipitated with UL70 but not UL44 (data not shown). Similar results were also observed in human foreskin fibroblasts infected with HCMV and uninfected HeLa, 293T, and U251 cells that were transfected with pCMV-UL70 and pCMV-UL44 (data not shown). These results suggest that DNAJB6 (a or b) may specifically interact with UL70 but not UL44 during HCMV infection.

### Mapping of the domain of DNAJB6 involved in the interaction with UL70

**Figure 2:**
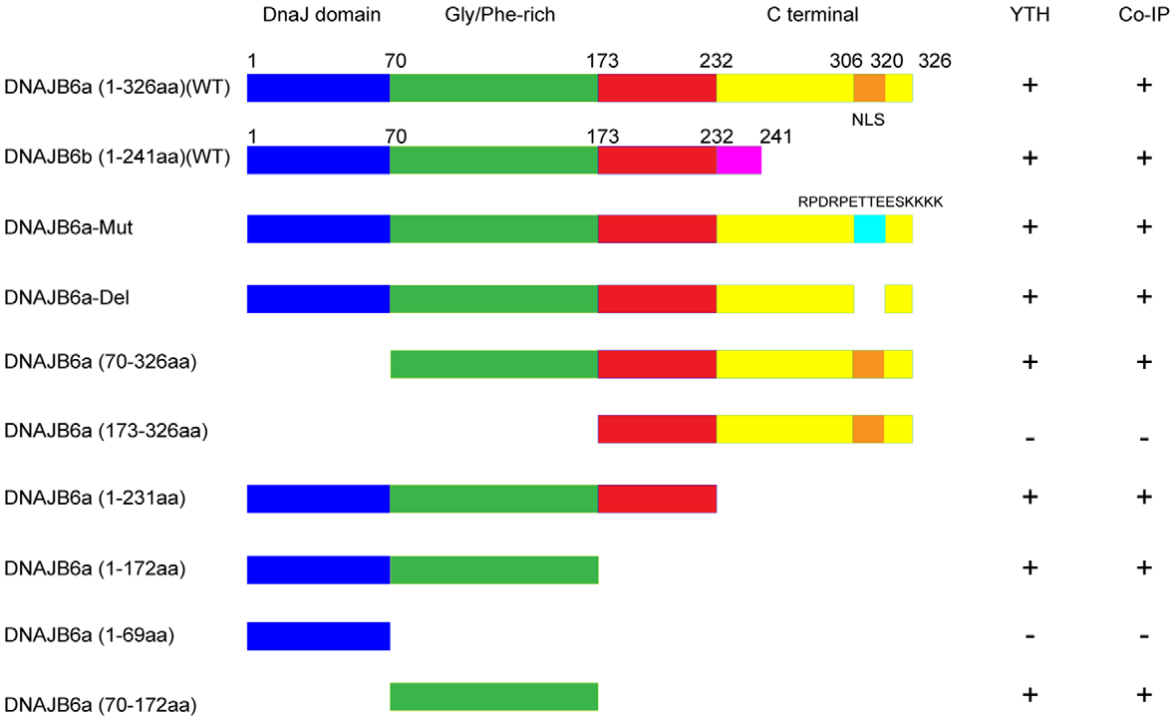
Schematic diagram of DNAJB6a and DNAJB6b and their deletion mutants that interact with UL70 as identified by the two hybrid screen in yeast (YTH) and co-immunoprecipitation (co-IP) in U251 cells. DNAJB6 (a and b) proteins share the N-terminal 231 amino acids, which include the highly conserved DnaJ homology region (in blue), a Gly/Phe-rich region (in green), and a linker region (in red) but differ at the less conserved C-terminal region (in yellow and pink for DNAJB6a and b, respectively) in which a NLS (in orange) is present in the longer DNAJB6a but is absent in the shorter DNAJB6b [38], [40]. The NLS was mutated and deleted in mutants DNAJB6a-Mut and DNAJB6a-Del, respectively. The interactions that were positive and negative in the two hybrid screen or co-IP were marked as “+” and “−”, respectively.

As a type II Hsp40/DNAJ chaperone protein, DNAJB6 (a or b) has three distinct domains, (i) a highly conserved J domain at the amino terminus, which is known to mediate interaction with Hsp70 and regulate its ATPase activity, (ii) a Gly/Phe-rich region which usually follows the J domain and may act as a flexible linker between the J domain and the C-terminal region, and (iii) a less conserved C-terminal domain that binds to its substrate and client protein (Figure 2) [29], [42]. DNAJB6a and DNAJB6b are identical in their N-terminal 231 amino acids but differ by 105 and 10 amino acids, respectively, in their C-termini [38]. To further investigate the association of UL70 and DNAJB6 and to map the domains of DNAJB6 required for this association, we constructed a series of truncation mutants of DNAJB6a and b (Figure 2, Table 1). Yeast two hybrid analyses were carried out to investigate the binary interactions of these DNAJB6 mutants and UL70. Furthermore, a series of mammalian expression constructs, which encoded the FLAG-tagged DNAJB6 truncation mutants, were generated (Figure 2, Table 1). These constructs were then co-expressed with the Myc-tagged UL70 in U251 cells, and the interactions between the Myc-tagged UL70 and different FLAG-tagged DNAJB6 mutants were examined in co-IP experiments (Figure S3, data not shown)(Supporting Information). The results, summarized in Figure 2, indicate that the minimal DNAJB6 mutant that binds to UL70 contains amino acid 70 to 172 that covers the Gly/Phe-rich domain (Figure S3, lanes 7–9), which is the common region found in both DNAJB6a and b and which is conserved among Hsp40/DNAJ proteins [29], [42].

**Table 1:** Plasmid constructs used in the study.

Plasmids	Description	Reference/source
pGADT7	cloning vector for protein expression fused with the GAL4 activation domain in yeast	Clontech
pGBKT7	cloning vector for protein expression fused with the GAL4 DNA-binding domain in yeast	Clontech
pCMV-Myc	cloning vector for protein expression fused with c-Myc tag in mammalian cell	Clontech
pCDNA3.1(+)	cloning vector for protein expression without tag in mammalian cell	Clontech
pCMV6-XL5-DNAJB6a	pCMV6-XL5 containing DNAJB6a cDNA full length sequence	OriGene
pCMV6-XL5-DNAJB6b	pCMV6-XL5 containing DNAJB6b cDNA full length sequence	OriGene
pGBKT7-UL70	pGBKT7 containing HCMV UL70 full length sequence	This study
pCMV-Myc-UL70	pCMV-Myc containing HCMV UL70 full length sequence	This study
pCMV-Myc-UL25	pCMV-Myc containing HCMV UL25 full length sequence	This study
pRK11-FLAG	cloning vector for protein expression fused with FLAG tag in mammalian cell	This study
pGADT7-DNAJB6a	pGADT7 containing DNAJB6a cDNA full length sequence (1–981 bp)	This study
pGADT7-DNAJB6a (70–326aa)	pGADT7 containing DNAJB6a cDNA truncated sequence (208–981 bp)	This study
pGADT7-DNAJB6a (173–326aa)	pGADT7 containing DNAJB6a cDNA truncated sequence (517–981 bp)	This study
pGADT7-DNAJB6a (1–231aa)	pGADT7 containing DNAJB6a cDNA truncated sequence (1–693 bp)	This study
pGADT7-DNAJB6a (1–69aa)	pGADT7 containing DNAJB6a cDNA truncated sequence (1–207 bp)	This study
pGADT7-DNAJB6a (1–172aa)	pGADT7 containing DNAJB6a cDNA truncated sequence (1–516 bp)	This study
pGADT7-DNAJB6a (70–172aa)	pGADT7 containing DNAJB6a cDNA truncated sequence (208–516 bp)	This study
pGADT7-DNAJB6b	pGADT7 containing DNAJB6b cDNA full length sequence (1–726 bp)	This study
pGADT7-DNAJB6a-Mut	pGADT7 containing the DNAJB6a cDNA sequence with the mutation at 306–320aa	This study
pGADT7-DNAJB6a-Del	pGADT7 containing the DNAJB6a cDNA sequence without 306–320aa	This study
pRK11-FLAG-DNAJB6a	pRK11-FLAG containing DNAJB6a cDNA full length sequence (1–981 bp)	This study
pRK11-FLAG-DNAJB6a (70–326aa)	pRK11-FLAG containing DNAJB6a cDNA truncated sequence (208–981 bp)	This study
pRK11-FLAG-DNAJB6a (173–326aa)	pRK11-FLAG containing DNAJB6a cDNA truncated sequence (517–981 bp)	This study
pRK11-FLAG-DNAJB6a (1–231aa)	pRK11-FLAG containing DNAJB6a cDNA truncated sequence (1–693 bp)	This study
pRK11-FLAG-DNAJB6a (1–69aa)	pRK11-FLAG containing DNAJB6a cDNA truncated sequence (1–207 bp)	This study
pRK11-FLAG-DNAJB6a (1–172aa)	pRK11-FLAG containing DNAJB6a cDNA truncated sequence (1–516 bp)	This study
pRK11-FLAG-DNAJB6a (70–172aa)	pRK11-FLAG containing DNAJB6a cDNA truncated sequence (208–516 bp)	This study
pRK11-FLAG-DNAJB6b	pRK11-FLAG containing DNAJB6b cDNA full length sequence (1–726 bp)	This study
pRK11-FLAG-DNAJB6a-Mut	pRK11-FLAG containing the DNAJB6a cDNA sequence with the mutation at 306–320aa	This study
pRK11-FLAG-DNAJB6a-Del	pRK11-FLAG containing the DNAJB6a cDNA sequence without 306–320aa	This study
pCMV-UL70	pCDNA3.1(+) containing HCMV UL70 full length sequence	This study
pCMV-UL44	pCDNA3.1(+) containing HCMV UL44 full length sequence	This study
pCMV-DNAJB6a	pCDNA3.1(+) containing the full length DNAJB6a cDNA sequence	This study
pCMV-DNAJB6b	pCDNA3.1(+) containing the full length DNAJB6b cDNA sequence	This study
pCMV-DNAJB6a-Mut	pCDNA3.1(+) containing the DNAJB6a cDNA sequence with the mutation at 306–320aa	This study
pCMV-DNAJB6a-Del	pCDNA3.1(+) containing the DNAJB6a cDNA sequence without 306–320aa	This study
pET-28a(+)-UL70	pET-28a(+) containing HCMV UL70 full length sequence	This study

### Cellular localization of UL70 and DNAJB6

**Figure 3:**
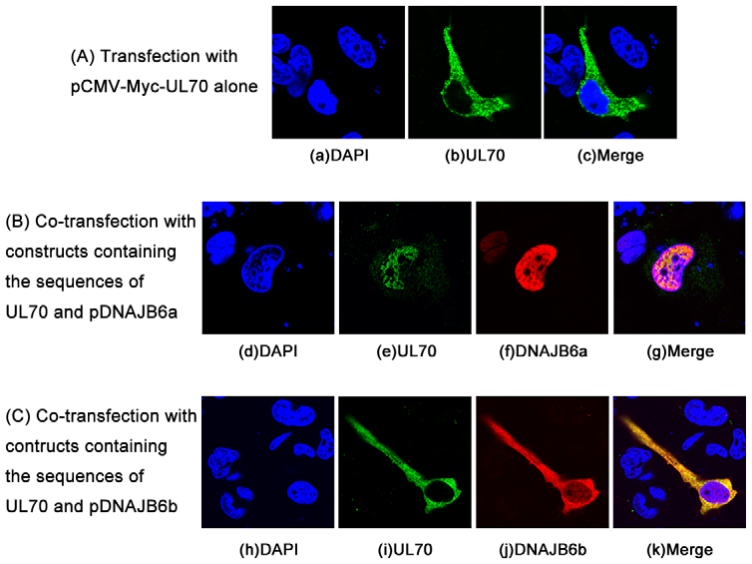
Co-localization of UL70 and DNAJB6a and DNAJB6b expressed in human cells. Cells were transfected with the construct containing the sequence of Myc-tagged UL70 alone (A) and in the presence of the constructs containing the sequences of FLAG-tagged DNAJB6 (a and b) (B and C), fixed at 48 hours posttransfection, stained with antibodies, and visualized using a microscope. The images of Myc-tagged UL70 (b, e, i), FLAG-tagged DNAJB6a (f) and DNAJB6b (j), and the nuclei stained with DAPI (a, d, h) were used to generate the composite images (c, g, k). The images show different levels of magnification.

If UL70 is associated with (or binds to) DNAJB6 in cells, it is expected that these proteins would localize within the same cellular compartments. As a result of alternative splicing, DNAJB6a and b proteins share the conserved N-terminal region but differ at the less conserved C-terminal region in which a NLS is present in the longer DNAJB6a, a protein predominantly localized in the nuclei, but is absent in the shorter DNAJB6b, a protein primarily localized in the cytoplasm [38], [39], [40]. To determine whether UL70 is co-localized with DNAJB6, cells were transfected with constructs expressing the FLAG-tagged DNAJB6a or b and Myc-tagged UL70, and the cellular localization of the expressed proteins was studied using immunofluorescence microscopy. In cells transfected with construct pCMV-Myc-UL70 alone, UL70 was found to be primarily expressed in the cytoplasm (Figure 3A). In co-transfection experiments, both the Myc-tagged UL70 and FLAG-tagged DNAJB6a were found to be predominately localized in the nuclei of the cotransfected cells while UL70 and DNAJB6b were primarily localized in the cytoplasm of the cotransfected cells (Figure 3B–C). Similar results were also observed in HeLa cells, 293T cells, and cells stained with antibodies against DNAJB6a or b (data not shown). Cells that had not been exposed to antiserum recognizing DNAJB6a or b, FLAG, or Myc epitopes showed no obvious fluorescence, indicating that the staining seen in cells is not due to a nonspecific cross-reaction of the secondary antibody with a viral or cellular protein. These observations are consistent with our results of the UL70-DNAJB6 interactions from the two-hybrid screens in yeast and the co-IP experiments in human cells.

To further confirm these results and investigate cellular localization of untagged UL70 and DNAJB6 proteins, cells were transfected with either pCMV-UL70 alone or in the presence of pCMV-DNAJB6a or pCMV-DNAJB6b, and then stained with anti-UL70 and anti-DNAJB6a or b antibodies. Similar to the results with the tagged proteins (Figure 3), in cells transfected with construct pCMV-UL70 alone, UL70 was found to be primarily expressed in the cytoplasm (Figure S4A)(Supporting Information). In co-transfection experiments, both the UL70 and DNAJB6a were found to be primarily localized in the nuclei while UL70 and DNAJB6b were predominately localized in the cytoplasm (Figure S4B–C). The cellular localization of DNAJB6 and UL44 was also studied to determine if the expression of DNAJB6 affects the localization of other HCMV replication proteins (Figure S5) (Supporting Information). In these experiments, cells were transfected with either pCMV-UL44 alone or in the presence of pCMV-DNAJB6a or pCMV-DNAJB6b, and then stained with anti-UL44 and anti-DNAJB6a or b antibodies. No difference in the cellular localization of UL44, which has previously been shown to be exclusively expressed in nuclei [1], was found in cells that were transfected with pCMV-UL44 alone or in the presence of pCMV-DNAJB6a and b (Figure S5A–C)(Supporting Information). Consistent with our results of the UL70-DNAJB6 interactions from the two-hybrid screens in yeast and the co-IP experiments in human cells, these results suggest that the tag sequences in Myc-UL70 and FLAG-DNAJB6 (a and b) do not affect the interaction and co-localization of UL70 with DNAJB6a and b, and that DNAJB6 may specifically interact with and affect the cellular distribution of UL70.

**Figure 4:**
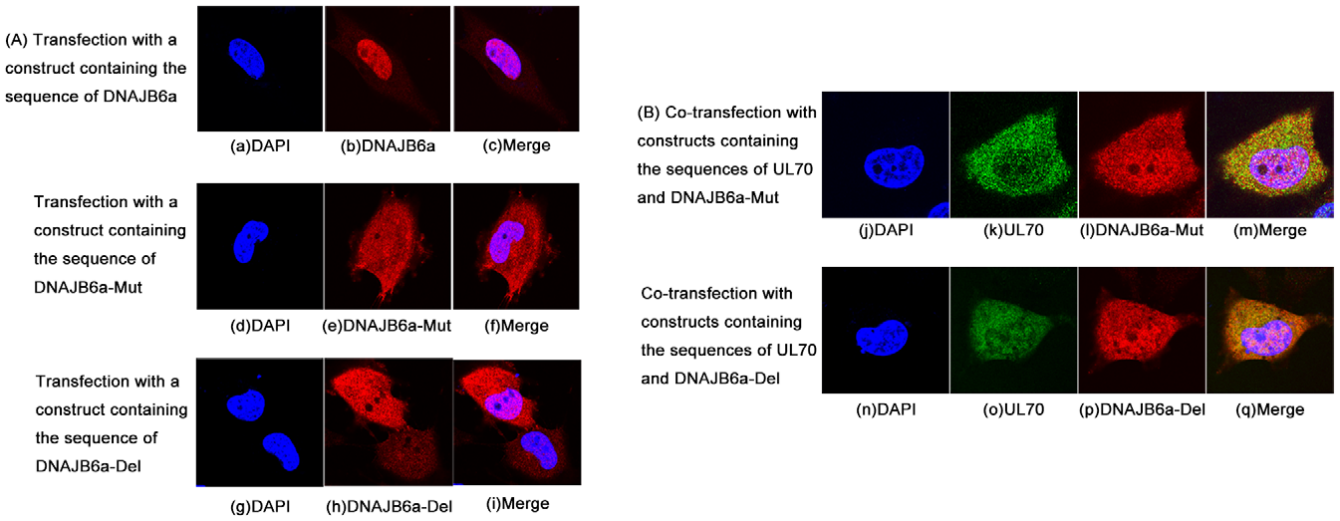
Role of the nuclear localization signal of DNAJB6 isoform a in the nuclear import of UL70. U251 cells were transfected with constructs pRK11-FLAG-DNAJB6a, pRK11-FLAG-DNAJB6a-Mut, and pRK11-FLAG-DNAJB6a-Del alone (A) and in the presence of construct plasmid pCMV-Myc-UL70 (B). At 48 hours posttransfection, cells were fixed, stained with antibodies, and visualized using a microscope. The images of Myc-tagged UL70 (k, o), FLAG-tagged DNAJB6a (b), DNAJB6a-Mut (e and l), and DNAJB6a-Del (h and p) and the nuclei stained with DAPI (a, d, g, j, and n) were used to generate the composite images (c, f, i, m, and q).

Previous studies have predicted the presence of a nuclear localization signal (NLS) in DNAJB6a from 306 to 320 amino acid residue (KRKKQKQREESKKKK), which is not present in DNAJB6b and is responsible for the nuclear localization of DNAJB6a [38], [40]. If the interaction of DNAJB6a with UL70 plays an important role in the cellular localization of UL70, it is expected that inactivation of the NLS may affect the ability of DNAJB6a to be localized in the nuclei, leading to a change in the cellular localization of UL70. To determine whether this is the case, we generated two DNAJB6a mutants, DNAJB6a-Mut and DNAJB6a-Del, in which the putative NLS was mutated to sequence RPDRPETTEESKKKK and was completely deleted, respectively (Figure 2). In cells transfected with constructs pRK11-FLAG-DNAJB6a, pRK11-FLAG-DNAJB6a-Mut, or pRK11-FLAG-DNAJB6a-Del alone, the mutant DNAJB6a proteins were found to be distributed throughout in both the cytoplasm and nuclei while the wild type protein was expressed primarily in the nuclei (Figure 4A). In co-transfection experiments, the Myc-tagged UL70 was found to be co-localized in both the cytoplasm and nuclei with the DNAJB6a mutant proteins (Figure 4B) but in the nuclei with the wild type DNAJB6a protein (Figure 3B). Together, these results further confirm the association between DNAJB6 and UL70 and suggest that the cellular localization of UL70 is affected by the presence of DNAJB6a and b, which are primarily localized in the nuclear and cytoplasmic compartment, respectively.

### Effects of up- and down-regulation of the expression of DNAJB6 on the cellular localization of UL70

UL70 is required to be in the nuclei, where viral DNA synthesis occurs during HCMV infection. Thus, our results that UL70 interacts with the nuclear DNAJB6a and cytoplasmic DNAJB6b protein and is primarily localized in the nuclei and cytoplasm raise the possibility that DNAJB6a and b may facilitate nuclear import of UL70 and sequester this protein in the cytoplasm, respectively. It is conceivable that overexpression of DNAJB6a and b, a predominantly nuclear and cytoplasmic protein, may lead to an accumulation of UL70 in the nuclei and cytoplasm, respectively.

**Figure 5:**
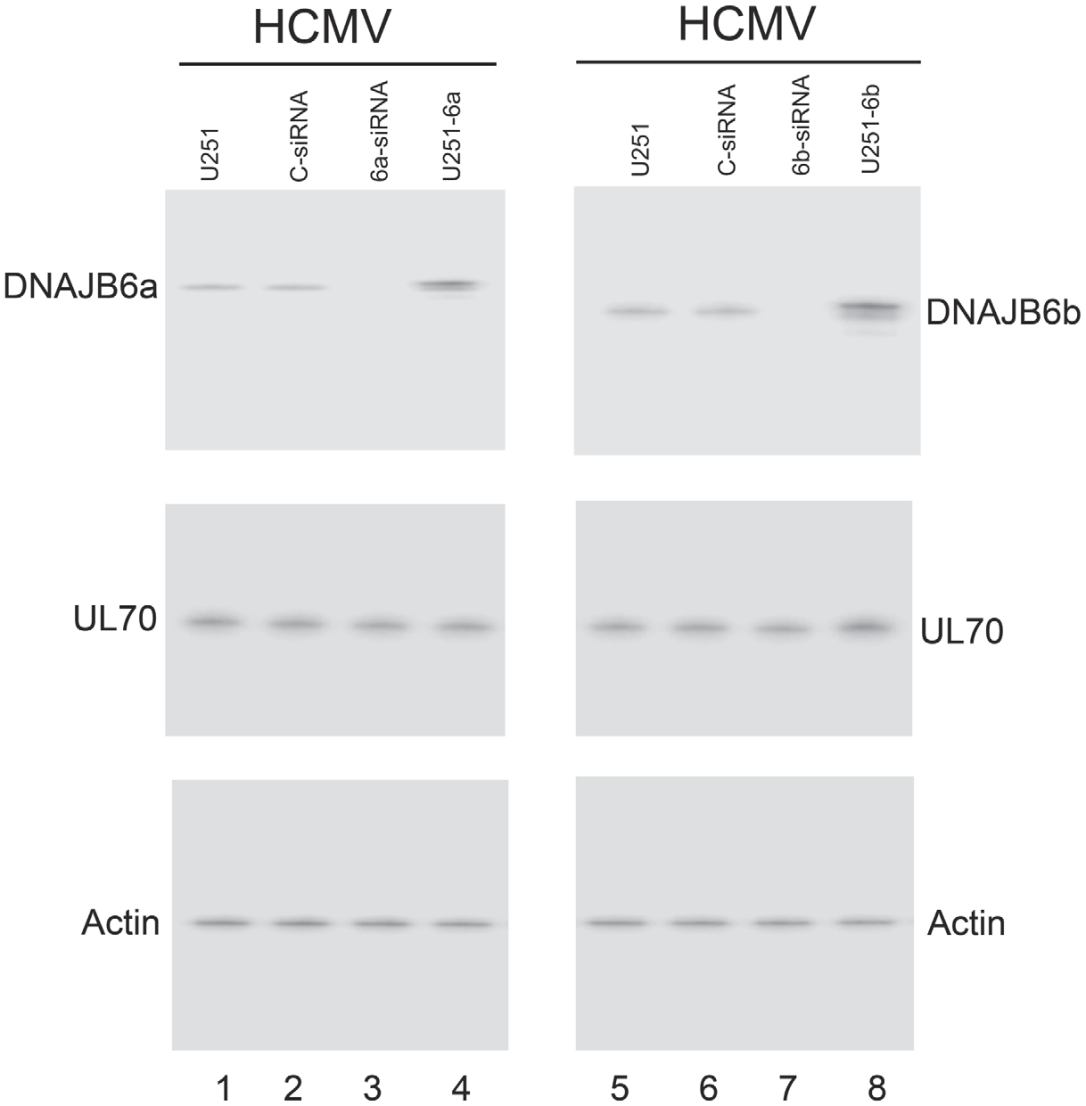
Up- and down-regulated expression of DNAJB6 in cells. Western blot analysis of the levels of DNAJB6a (lanes 1–4) and b (lanes 5–8) and Myc-tagged UL70 in the parental U251 cells (U251) (lanes 1 and 5), the DNAJB6a-expressing U251-6a (U251-6a) (lane 4) and DNAJB6b-expressing cells (U251-6b) (lane 8), or U251 cells that were transfected with anti-DNAJB6a siRNA (6a-siRNA) (lane 3), anti-DNAJB6b siRNA (6b-siRNA) (lane 7), or control siRNA (C-siRNA) (lanes 2 and 6). The expression of cellular actin was used as the internal control. Cells were transfected with pCMV-Myc-UL70 in the absence and presence of siRNAs. Forty-eight hours after transfection, cells were infected with HCMV at MOI of 1. Protein samples were prepared at 48–72 hours postinfection. The membranes were reacted with antibodies, stained using a Western chemiluminescent substrate kit (GE Healthcare), and quantitated with a STORM840 PhosphorImager (GE Healthcare) or a Gel Documentation Station (BioRad, Hercules, CA) [54], [55]. A dilution series of the samples was analyzed and the results were compared in order to accurately determine the protein levels. Quantitation was performed in the linear range of protein detection [54], [55].

To study the effect of overexpression of DNAJB6 on the cellular localization of UL70, we constructed stable U251 cell lines, U251-6a and U251-6b, which constitutively expressed DNAJB6a and DNAJB6b, respectively, and a control cell line, U251-C, which contained the empty expression vector. Western blotting of HCMV-infected cell lysates indicated a ∼5 fold elevated level of DNAJB6a and b in U251-6a and U251-6b cells compared to the parental U251 and control U251-C cells (Figure 5, lanes 1, 4, 5, and 8). The constructed cell lines and the parental U251 cells were indistinguishable in terms of their growth and viability for three months (data not shown), suggesting that the over-expression of DNAJB6 did not result in significant cytotoxicity.

**Figure 6:**
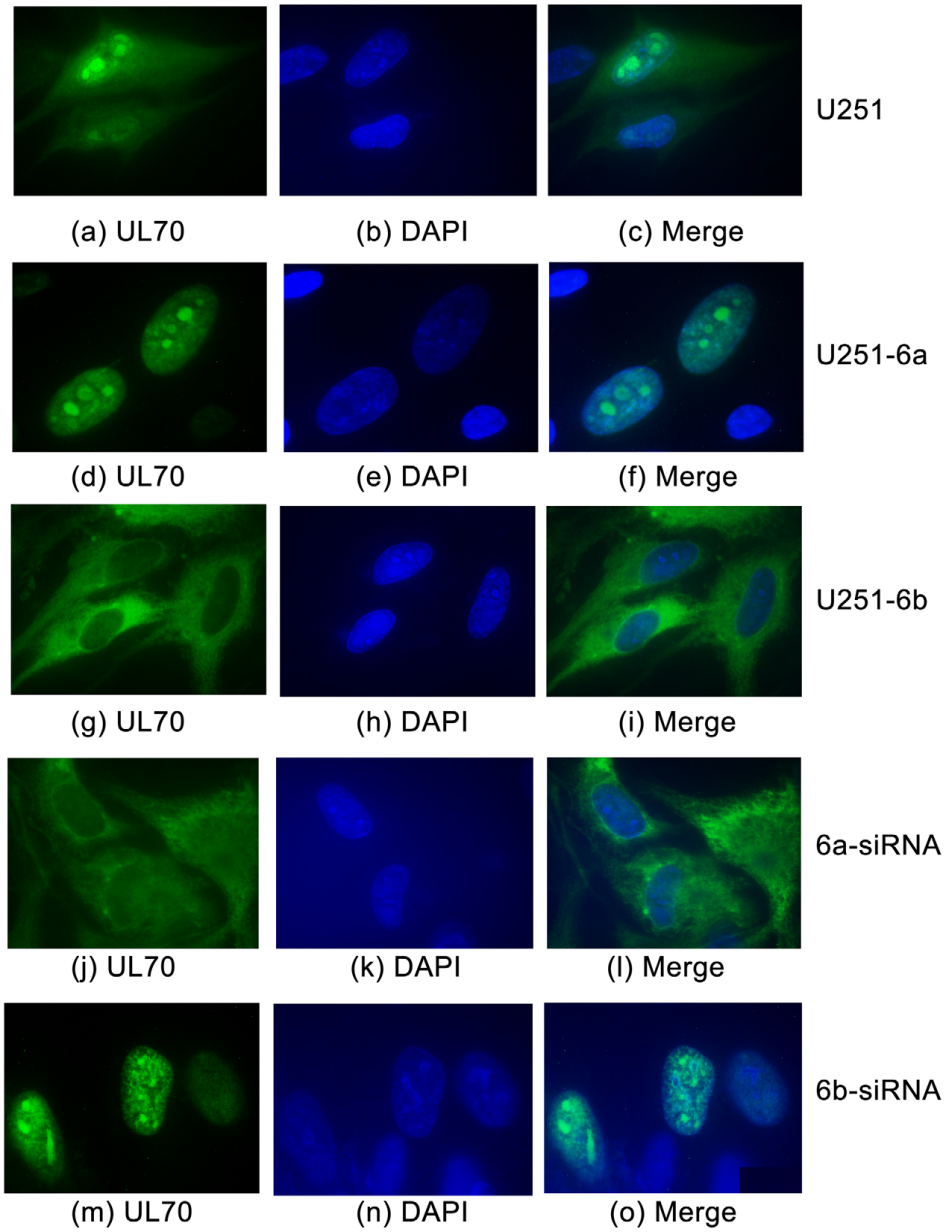
Effect of up- and down-regulation of the expression of DNAJB6 on the cellular distribution of UL70. Immunofluorescence microscopy of the cellular localization of UL70 in the parental U251 cells (U251), cells overexpressing DNAJB6a (U251-6a) and b (U251-6b), or the cells treated with anti-DNAJB6a (6a-siRNA) and anti-DNAJB6b siRNA (6b-siRNA). Cells were infected with HCMV (MOI = 5) at 48 hours posttransfection, fixed at 48–72 hours postinfection, stained with antibodies, and visualized. The images of Myc-tagged UL70 (green) (a, d, g, j, m) and the nuclei stained with DAPI (blue) (b, e, h, k, n) were used to generate the composite images (c, f, i, l, o). The images show different levels of magnification.

To determine the localization of UL70 in HCMV-infected cells, the constructed cell lines were transfected with pCMV-Myc-UL70 and then infected with HCMV. An MOI of 1–5 was used to assure that most cells were infected. Indeed, IE1 expression was detected in a majority of the cells when cells were stained with an anti-IE1 antibody (data not shown). HCMV infection appeared to promote nuclear import of UL70 in U251 cells as a substantial amount of UL70 signal was found in the nuclei at 48–72 hours postinfection, compared to the uninfected cells (Figure 6, a–c, compared to Figure 3A, a–c). In the presence of HCMV infection, the localization of UL70 in the DNAJB6-expressing U251-6a and -6b cells was significantly different from that in the parental U251 cells and the control U251-C cells. At 48 hours postinfection, UL70 was primarily found in the nuclei of U251-6a cells (Figure 6, d–f) and the cytoplasm of U251-6b cells, respectively (g–i). In contrast, both nuclear and cytoplasmic expression of UL70 was found in the U251 (Figure 6, a–c) and U251-C cells (data not shown). Counting the cells in these experiments suggest that UL70 was found in the nuclei of ∼95% of U251-6a cells and the cytoplasm of ∼96% of U251-6b cells, respectively, while the remaining ∼5% U251-6a and ∼4% U251-6b cells as well as ∼98% of U251 and U251-C cells showed UL70 in both the cytoplasm and nucleus (Table S1 in Text S1)(Supporting Information). Similar results were also observed when cells were transfected with pCMV-UL70 instead of pCMV-Myc-UL70 and stained with the anti-UL70 antibody (Table S1 in Text S1), consistent with our observations that the tag sequence does not affect the interaction and co-localization of UL70 with DNAJB6 (Figure 1 and 3, S1, S2, and S4, S5). These results suggest that overexpression of DNAJB6a and b leads to an accumulation of UL70 in the nuclei and cytoplasm, respectively.

As overexpression of DNAJB6a and b leads to the nuclear and cytoplasmic accumulation of UL70, it is conceivable that down-regulation of DNAJB6a and b expression may promote cytoplasmic accumulation and nuclear import of UL70, respectively. To determine whether this is the case, U251 cells were co-transfected with pCMV-Myc-UL70 and siRNA molecules that were designed either to recognize various regions of DNAJB6a and b mRNA (6a-siRNA and 6b-siRNA) or not to recognize any viral or cellular transcripts (control siRNA or C-siRNA), and then either mock-infected or infected with HCMV at 48 hours post-transfection. The 6a-siRNA molecules were a pool of three siRNAs that targeted different unique regions of the DNAJB6a mRNA while the 6b-siRNA molecules were a pool of two siRNAs that targeted different unique regions of the DNAJB6b mRNA (Table S2 in Text S1)(Supporting Information). Down-regulation of DNAJB6a and b expression mediated by siRNA has been shown to have no effect on cell viability [39], [45]. Western blotting of transfected cell lysates indicated that the level of DNAJB6a and b proteins was reduced by more than 80% in cells transfected with 6a- and 6b-siRNA compared to that in cells transfected with control C-siRNA (Figure 5, lanes 2, 3, 6, and 7). In the presence of HCMV infection, the localization of UL70 in cells treated with 6a- and 6b-siRNA was predominantly cytoplasmic and nuclear, respectively, while both nuclear and cytoplasmic expression of UL70 was found in the parental U251 cells and the control-siRNA treated cells (Figure 6, j–o, data not shown). Counting the cells in these experiments suggested that UL70 was found in the nuclei of ∼92% 6b-siRNA-treated cells and the cytoplasm of ∼93% 6a-siRNA-treated cells, respectively, while the remaining ∼8% 6b-siRNA-treated cells and ∼7% 6a-siRNA-treated cells as well as ∼98% of U251 and control-siRNA-treated cells showed UL70 in both the cytoplasm and nucleus (Table S1 in Text S1)(Supporting Information). Similar results were also observed when cells were transfected with pCMV-UL70 instead of pCMV-Myc-UL70 and stained with the anti-UL70 antibody (Table S1 in Text S1). These results suggest that down-regulation of the expression of DNAJB6a and b reduces the nuclear and cytoplasmic distribution of UL70, respectively.

**Figure 7:**
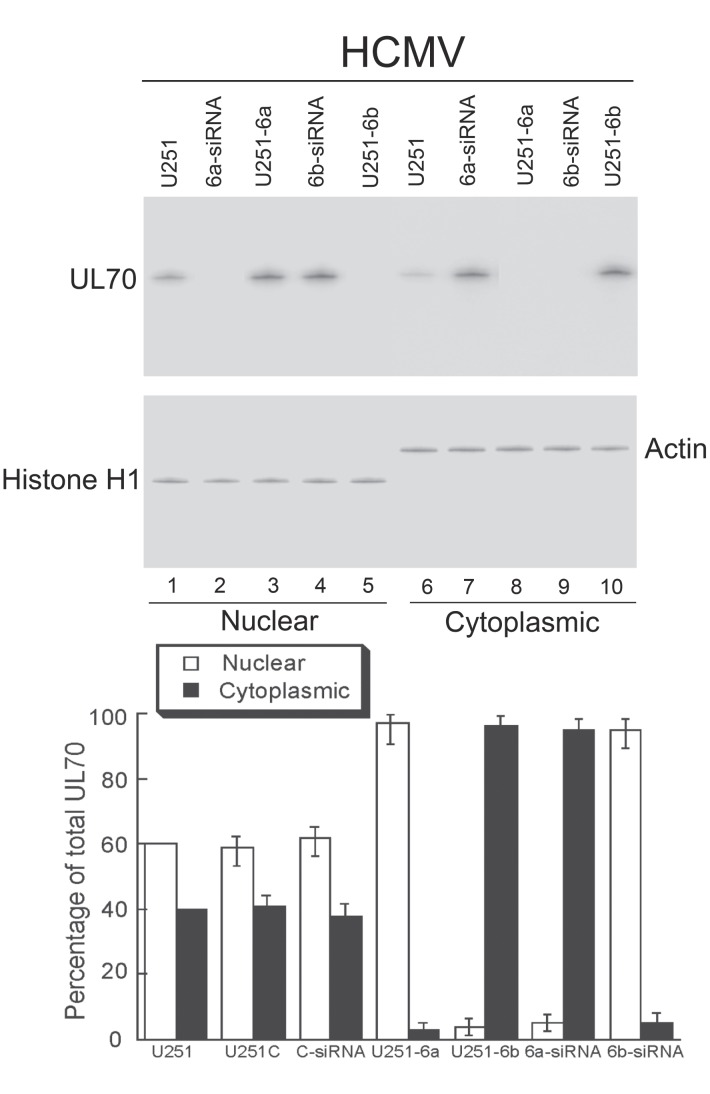
Effect of up- and down-regulation of the expression of DNAJB6 on the distribution of UL70 in nuclear and cytoplasmic fractions. Different cells (e.g. parental U251 cells, U251-6a and U251-6b cells, and 6a- and 6b-siRNA treated cells) were transfected with pCMV-Myc-UL70 in the absence and presence of siRNAs. At 48 hours posttransfection, cells were infected with HCMV (MOI = 1). At 48–72 hours postinfection, cells were harvested and separated into nuclear and cytoplasmic fractions. Equivalent amounts of each fraction were analyzed by immunoblotting with anti-Myc. The purity of the nuclear and cytoplasmic fractions was assayed by immunoblotting with anti-histone H1 and anti-Actin, respectively. The membranes were reacted with antibodies, stained using a Western chemiluminescent substrate kit (GE Healthcare), and quantitated with a STORM840 PhosphorImager (GE Healthcare) or a Gel Documentation Station (BioRad, Hercules, CA) [54], [55]. A dilution series of the samples was analyzed and the results were compared in order to accurately determine the protein levels. Quantitation was performed in the linear range of protein detection [54], [55]. The experiments were in duplicate and repeated three times. The standard deviation is indicated by the error bar (lower panel).

To confirm the effects of DNAJB6 expression on the localization of UL70, we determined the distribution of UL70 by cell fractionation and Western blot analysis (Figure 7). Different cells (e.g. U251-C, U251-6a and -6b cells, and cells treated with C-siRNA or 6a- or 6b-siRNA) were first transfected with pCMV-Myc-UL70, then infected with HCMV, and subjected to differential centrifugation into nuclear and cytoplasmic fractions. The purity of the fractions was confirmed by immunoblotting for histone H1 (nuclear marker) and actin (cytoplasmic marker) (Figure 7). In parental U251 cells that were transfected with pCMV-Myc-UL70 and infected with HCMV, 60% of UL70 was found in the nuclei while 40% was in the cytoplasm (Figure 7). Similar results were also found in control cells that either contained the empty expression vector (U251-C cells) or were treated with siRNA molecules designed not to recognize any viral or cellular transcripts (C-siRNA)(Figure 7, lower panel). However, down-regulation of the expression of DNAJB6a and b resulted in a decrease (less than 5%) and increase (more than 95%) in the level of UL70 in the nuclear fractions in the 6a- and 6b-siRNA treated cells, respectively. In contrast, more than 95% and less than 5% of UL70 was found in the nuclear fractions in cells over-expressing DNAJB6a and b (i.e. U251-6a and -6b cells), respectively (Figure 7). Similar results were also observed when cells were transfected with pCMV-UL70 instead of pCMV-Myc-UL70 and stained with the anti-UL70 antibody (Figure S6)(Supporting Information), consistent with our observations that the tag sequence does not affect the interaction and co-localization of UL70 with DNAJB6. These observations confirm our results from the immunofluorescence microscopy experiments and suggest that DNAJB6 plays an important role in the cellular localization of UL70.

### Effect of up- and down-regulation of the expression of DNAJB6 on HCMV DNA synthesis and lytic infection

It is expected that a change of the UL70 level in the nucleus as a result of altered expression of DNAJB6 may affect the level of HCMV DNA synthesis and virus production. This is because UL70 is believed to encode the viral primase essential for HCMV DNA replication, which occurs in the nuclei [1]. To determine the effect of altered expression of DNAJB6 on HCMV infection, we examined the profiles of viral gene expression by assaying the production of viral proteins of different kinetic classes during replication in cells where the expression of DNAJB6 had been altered.

**Figure 8:**
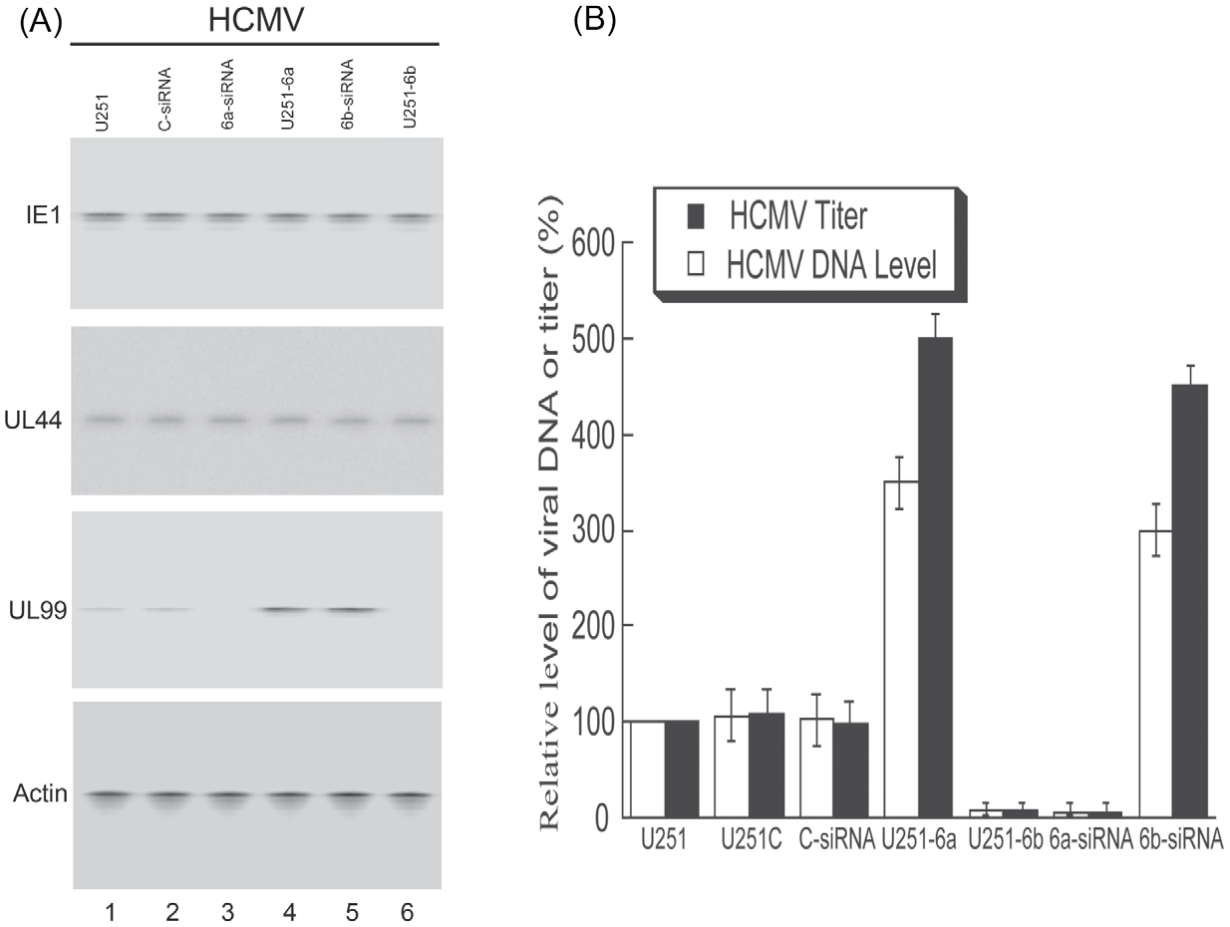
Effect of up- and down-regulation of the expression of DNAJB6 on HCMV gene expression, and viral genomic synthesis and production. (A) Western blot analysis of the levels of HCMV IE1, UL44, and UL99 in the parental U251 cells (U251) (lane 1), cells overexpressing DNAJB6a (U251-6a) (lane 4) and b (U251-6b) (lane 6), or U251 cells that were transfected with either anti-DNAJB6a (6a-siRNA) (lane 3) and DNAJB6b siRNA (6b-siRNA) (lane 5) or control siRNA (C-siRNA) (lane 2). The expression of cellular actin was used as the internal control. At 48 hours posttransfection, cells were infected with HCMV (MOI = 1). Protein samples were prepared at 48–72 hours postinfection. (B) To assay the level of intracellular viral DNA, cells were harvested at 72 hours postinfection. To assay the level of viral production, total infection cultures were collected at 5 days postinfection and viral titers were determined. The values of the relative levels of HCMV DNA and titers, which are the means from triplicate experiments, represent the ratios of the levels of viral DNA or titers in different cells to those in the parental U251 cells (U251), respectively. The analyses were repeated three times and the standard deviation is indicated by the error bar.

Different cells (e.g. U251-C and U251-6a and -6b cells, and cells treated with C-siRNA or 6a- or 6b-siRNA) were infected with HCMV and then harvested at several time points postinfection. Protein levels were assayed by Western blot analysis. At 48–72 hours postinfection, a reduction of more than 80% in the expression of DNAJB6a and b was found in cells treated with 6a- and 6b-siRNA molecules, while the levels of DNAJB6a and b in U251-6a and -6b cells were about 5-fold higher than those in U251 cells (Figure 5, data not shown). The up- and down-regulation of the expression of DNAJB6 did not appear to affect the protein levels of either the immediately early viral gene product IE1 or the early gene product UL44 (Figure 8A). However, a decrease in the protein level of UL99 was detected in either the DNAJB6b-expressing U251-6b cells or cells treated with 6a-siRNA while an increase in the level of UL99 was found in either the DNAJB6a-expressing U251-6a cells or cells treated with 6b-siRNA molecules (Figure 8A). UL99 is the product of a viral late gene, whose expression is dependent upon viral DNA synthesis [1]. We speculated that the change of UL99 levels in these cells might be an effect of modulation of viral DNA synthesis, as a result of a change of nuclear UL70 level due to altered expression of DNAJB6a and b.

Two sets of experiments were carried out in these cells to determine whether this is the case. In the first set of experiments, total intracellular DNAs were isolated and the levels of viral DNA synthesis were assayed. The number of viral genomes were quantified by quantitative real-time PCR (qPCR) and determined by normalizing the copy number of the viral UL83 DNA sequence to the copy number of the cellular β-actin gene sequence. At 72 hours postinfection, a reduction of more than 90% in the number of copies of UL83/β-actin was observed in either the DNAJB6b-expressing U251-6b cells or cells treated with anti-DNAJB6a 6a-siRNA molecules while an increase of about 3-fold was found in either the DNAJB6a-expressing U251-6a cells or cells treated with anti-DNAJB6b 6b-siRNA molecules (Figure 8B). In contrast, we observed no significant difference in the number of copies of UL83/β-actin among the parental U251 cells, the empty expression vector-containing U251-C cells, and the control C-siRNA treated cells (Figure 8B). These results suggest that up- and down-regulation of DNAJB6 expression alters the level of HCMV DNA synthesis, respectively.

In the second set of experiments, viral progeny production in these cells was determined. Virus stocks were prepared from the infected cultures (cells and culture medium together) and their titers were determined. At 5 days after infection, a reduction of at least 20-fold in viral yield was observed in either the DNAJB6b-expressing U251-6b cells or cells treated with 6a-siRNA while an increase of about 5-fold was found in either the DNAJB6a-expressing cells or cells treated with 6b-siRNA molecules (Figure 8B). In contrast, we observed no significant difference in viral yield among the parental U251 cells, the empty expression vector-containing U251-C cells, and the control C-siRNA treated cells (Figure 8B). These results suggest that altered expression of DNAJB6 modulates viral DNA replication and production.

## Discussion

Genomic DNA replication is a universal and essential process for herpesvirus infection [1], [46]. Each component of the DNA replication machinery, including the primase-helicase complex, which in HCMV consists of UL70, UL102, and UL105, is highly conserved among all herpesviruses [6], [13], [14], [47], [48]. Understanding the potential interactions of these viral proteins as well as their interactions with human proteins is important for elucidating the mechanism of herpesvirus genomic DNA synthesis and for developing novel strategies for the treatment and prevention of herpesvirus infections. In this study, we provide the first direct evidence that HCMV UL70 protein specifically interacts with human Hsp40 proteins DNAJB6 (a and b). This interaction was identified by our results from the two-hybrid screen in yeast and co-immunoprecipitation in human cells. We also showed that the level of UL70 in the nuclei, the site of viral DNA synthesis, is increased in either cells overexpressing DNAJB6a or those in which DNAJB6b expression is down-regulated with anti-DNAJB6b siRNA molecules. Furthermore, the level of UL70 in the nuclei is reduced in either cells overexpressing DNAJB6b or those in which DNAJB6a expression is down-regulated with siRNA molecules specifically recognizing the DNAJB6a mRNA transcript. Finally, the level of viral DNA synthesis and progeny production is positively correlated with the levels of DNAJB6a protein and negatively correlated with those of DNAJB6b protein. These results suggest that DNAJB6a and b function to bind to UL70 and increase and reduce its nuclear import and its availability for viral DNA synthesis, leading to an increase and decrease of viral DNA replication and particle production, respectively.

As a chaperone protein, DNAJB6 may non-specifically bind to proteins that contain unnatural tagged sequences or are over-expressed. It is possible that DNAJB6 (a and b) interacts with the over-expressed and tagged UL70 protein but not the native viral protein. However, several lines of evidence presented in our study suggest that this is unlikely and that the results obtained in experiments using the tagged UL70 protein may be identical to those using the native untagged protein. First, DNAJB6 was co-immunoprecipitated with the Myc-tagged UL70 but not UL25, suggesting that DNAJB6 does not interact with the tag sequence (Figure S1). Second, DNAJB6 was co-immunoprecipitated in HCMV-infected cells with untagged native UL70 but not UL44, a HCMV replication protein, implying that DNAJB6 is not associated with UL44 but may specifically interact with UL70 during HCMV infection (Figure S2). Third, there is no difference in the cellular localization of the tagged and untagged UL70 protein with DNAJB6 (Figure 3 and S4). Fourth, there is no difference in cellular distribution of the tagged and untagged UL70 protein in cells expressing different levels of DNAJB6 (Figure 7 and S6, Table S1 in Text S1). These results suggest that (1) DNAJB6 may specifically interact with UL70, (2) that the tag sequence does not affect the interaction and co-localization of UL70 with DNAJB6, and (3) that the results obtained in experiments with the tagged UL70 protein may truly reflect those results obtained with the native untagged protein during HCMV infection.

Proteins from the families of heat shock proteins (Hsps), such as Hsp90, Hsp70, and Hsp40, can bind to diverse cellular targets and play critical roles in many essential cellular processes [27], [28]. Several Hsps have been shown to be important for the replication of viruses [29], [30], [31], [32], [33]. For example, Weller and colleagues first described the formation of chaperone-enriched domains in the nuclei of cells lytically infected with HSV-1 and further showed that Hsp90 is required for the nuclear import of the HSV-1 polymerase [30], [34]. A human Hsp40 protein has also been shown to interact with the HSV-1 origin-binding protein, UL9, and facilitate viral DNA replication [31], [49]. During KSHV infection, Hsp90β and DNAJB11 interact with viral K1 protein and are required for its anti-apoptotic activity [35] while the EBNA3A protein of EBV interacts with key components of an active Hsp70/Hsp40 chaperone complex [33]. DNAJB6, which is a class II DNAJ/heat shock protein (Hsp) 40 family protein, is highly conserved and ubiquitously expressed including in brain [29], [36], [37], [38], [39]. In neurons, over-expressed DNAJB6 effectively suppressed the aggregation of toxic proteins such as disease-associated polyglutamine protein, leading to the reduction of caspase activity and cellular toxicity [36], [50]. Furthermore, DNAJB6 has been shown to be essential for murine placental development [36]. These results highlight the important function of DNAJB6 in cell viability and development.

While numerous Hsp40 proteins have been shown to play key roles in the life cycles of viruses [29], little is known about the role of DNAJB6 in infections of viruses except HIV-2. It has been shown that DNAJB6 specifically interacts with the HIV-2 Vpx protein [45]. This interaction enhances the nuclear localization of Vpx and promotes nuclear import of viral pre-integration complexes. However, the specific mechanism for nuclear import of Vpx is not yet defined, and it is not clear in this case whether DNAJB6 is displaying autonomous chaperone activity or acting to stimulate Hsp70 activity. It has not been reported that DNAJB6 interacts with any proteins encoded by herpesviruses. How DNAJB6 interacts with UL70 is currently unknown. Our results suggest that the binding of DNAJB6a and 6b promotes the nuclear import and cytoplasmic accumulation of UL70, leading to an enhancement and inhibition of HCMV DNA synthesis and particle production, respectively. While increasing DNAJB6a expression is clearly beneficial to CMV lytic infection, it will be interesting to determine whether the inhibition of HCMV replication mediated by DNAJB6b interaction with UL70 construes an antiviral defense for the benefit of the host or represents a means of viral temperance [43] for the benefit of HCMV, which is well known for its ability of a highly controlled replication during persistent and latent infections. It is conceivable that controlling the differential expression of DNAJB6 proteins, mediated by alternative splicing, may represent a novel mechanism for the regulation of HCMV replication and infection, by modulating the cellular localization of UL70.

Our findings of the DNAJB6-UL70 interaction may have general implications regarding the pathways for localization and assembly of the components of DNA replication machinery of herpesviruses. Each component of the tripartite helicase-primase complex is highly conserved in all herpesviruses [1], [46], [51]. For example, this complex is encoded by BBLF (helicase), BSLF1 (primase), and BBLF2/3 (primase-associated factor) in EBV; by UL52 (helicase), UL5 (primase), and UL8 (primase-associated factor) in HSV-1; and by ORF44 (helicase), ORF56 (primase), and ORF40/41 (primase-associated factor) in KSHV [6], [13], [14], [47], [48]. Proper localization of each component of the helicase-primase complex in the nuclei where viral DNA synthesis occurs is essential for viral infection, and its process is believed to be conserved among all herpesviruses [1]. In HSV-1, when expressed individually in human cells, UL5, UL8, and UL52 were localized in the cytoplasm [13], [15], [52]. However, when all three were expressed in the same cells, these proteins were localized in the nuclei. Similar results were also observed in the localization of BBLF, BSLF1, and BBLF2/3 of EBV [14]. In KSHV, when expressed individually in cells, ORF56 (primase), ORF44, and ORF40/41 were localized in the cytoplasm [47]. However, these three proteins were only localized in the nuclei when they were co-expressed with other core components of the viral DNA replication machinery including the DNA polymerase, the polymerase processivity factor, and single strand DNA binding protein [47]. UL70 has been shown to interact with UL102 and UL105 [9]. However, little is currently known about how UL70 (and its counterparts in other viruses) interacts with UL102 and UL105 (and their counterparts in other viruses) to achieve proper localization in the nuclei. No cellular factors that facilitate the nuclear import of these proteins by interaction with them have been reported. Our results showed that UL70, when expressed alone in human cells, is exclusively localized in the cytoplasm. This is consistent with the cytoplasmic localization pattern of its counterpart in HSV, EBV, and KSHV when these proteins were expressed alone in the absence of other viral proteins [14], [52], [53]. Furthermore, we showed for the first time that UL70 specifically interacts with DNAJB6a and b, which are predominantly localized in the nuclei and cytoplasm, respectively. DNAJB6a and b may play a key role in the cellular localization of UL70 by binding to and partitioning this protein in the nuclear and cytoplasmic compartment. Whether DNAJB6 also interacts with the counterparts of UL70 in other viruses is currently unknown. Our results indicate that the minimal DNAJB6 mutant that binds to UL70 contains amino acid 70 to 172, which are shared by both DNAJB6a and b and which are conserved among Hsp40/DNAJ proteins [12]. It will be interesting to determine whether the common and conserved regions of DNAJB6a and b also interact with the primase proteins found in other herpesviruses.

It is possible that DNAJB6 interacts with HCMV proteins other than UL70, leading to a modulation of viral DNA replication and infection as observed in our experiments. However, yeast two hybrid screen experiments in our laboratory revealed no positive interaction between DNAJB6 and more than 100 HCMV ORFs other than UL70 [44] (data not shown). Co-IP experiments using antibodies, which are against several HCMV proteins and are currently commercially available (e.g. UL44, IE1, and UL83), did not reveal any positive interactions either (Figure S2, data not shown). While we can not completely exclude the possibility that DNAJB6 may affect the HCMV DNA replication and infection by interacting with HCMV proteins other than UL70, our results that modulation of the expression of DNAJB6 leads to the same changes in the cellular localization of UL70 in mock-infected cells as in HCMV-infected cells (e.g. Figure 3, Figure 6, Figure S4, and Table S1 in Text S1) suggest that DNAJB6 may interact with UL70 and alters its cellular localization in the absence of other HCMV proteins, possibly leading to a modulation of viral DNA replication during HCMV infection.

Using several different types of cells, including U251, 293T, and HeLa cells, we showed that UL70 potentially interacts with DNAJB6 in co-IP experiments. Furthermore, the roles of DNAJB6 in UL70 localization and HCMV infection were investigated using U251 cells. U251 cells, which are derived from neuronal tissues infected naturally with HCMV in vivo, are permissive to HCMV replication and have been used to study HCMV infection [1]. It may be important to test the role of DNAJB6 in fibroblasts that are fully permissive and commonly used to study HCMV infection. However, it is technically difficult to carry out similar experiments in fibroblasts as the poor efficiency of transfection in these cells resulted in low level of UL70 and anti-DNAJB6 siRNA. Moreover, fibroblasts are primary cells and therefore, have a limited number of passages. It is difficult to generate a fibroblast cell line that constitutively and stably expresses high levels of DNAJB6 after substantial number of passages. In contrast, U251 cells are immortalized and therefore, we can easily generate U251 cell lines that constitutively express stable and high levels of DNAJB6. Further experiments in fibroblasts and other cells permissive to HCMV infection should elucidate the role of DNAJB6 in these cells.

Our results indicate that overexpression of DNAJB6b and downregulation of expression of DNAJB6a reduces the nuclear import of UL70 and increases its accumulation in the cytoplasm. The mechanism of how UL70 is retained in the cytoplasm is currently unknown. It is conceivable that DNAJB6b sequesters UL70 in specific cytoplasmic compartments so that UL70 is not available for interaction with other viral or cellular protein carriers for nuclear transport. This is consistent with our recent observations that a cellular cytoplasmic protein Snapin is associated with and co-localizes with UL70 in the cytoplasm [41]. In the same cultured cell system, overexpression of Snapin protein increases the accumulation of UL70 in the cytoplasm and reduces its nuclear import, while down-regulation of the expression of Snapin reduces cytoplasmic accumulation of UL70 and increases its nuclear import [41]. These results suggest that UL70 can be sequestered in the cytoplasm by interacting with multiple cellular proteins. Meanwhile, overexpression of DNAJB6a and down-regulation of the expression of DNAJB6b increases nuclear import of UL70 and decreases its cytoplasmic accumulation. These observations suggest that the proper cellular localization of UL70 in either the cytoplasm or nucleus is at a delicate balance and may be dictated by the cellular expression level of DNAJB6a and b. It is conceivable that DNAJB6a functions as a specific cellular carrier for nuclear transport and competes with DNAJB6b for binding of UL70. When DNAJB6b expression is reduced, binding of UL70 to DNAJB6a is favored, leading to increased nuclear import of UL70. In contrast, DNAJB6b, when overexpressed, interacts with and sequesters UL70, leading to its accumulation in the cytoplasm. This hypothesis is supported by our observations that up- and down-regulation of DNAJB6a promotes nuclear and cytoplasmic accumulation of UL70, respectively. It will be interesting to determine whether the cellular localization of the homologues of UL70 in other viruses also employ similar cellular pathways. These and future studies on the interactions of the herpesvirus DNA replication proteins with other viral and cellular proteins will provide significant insight into the mechanism of proper localization and assembly of the helicase-primase complex during lytic infection of herpesviruses.

## Materials and Methods

### Ethics statement

This study was carried out in strict accordance with the recommendations in the Guide for the Care and Use of Laboratory Animals of the National Institutes of Health. The protocol for all animal experiments was either approved by the Animal Care and Use Committee of the University of California-Berkeley (Protocol #R240) or by the Animal Care and Use Committee of the State Key Laboratory of Virology, Wuhan University (Wuhan, China). All efforts were made to minimize suffering.

### Plasmid constructs

The constructs generated in this study are listed in Table 1. The constructs containing the DNA sequences of DNAJB6 isoform a and b were purchased from Origene, Inc. (Rockville, MD). To generate constructs pGADT7-DNAJB6b and pRK11-FLAG-DNAJB6b used for expression in yeast and in human cells, the coding sequence of DNAJB6 isoform b (DNAJB6b) was amplified using the primers DNAJB6b-F (5′-TTGGCCATGGAGGCCGCGGCCGCGATGGTGGATTACTATGAA-3′) and DNAJB6b-R (5′-AACGAATTCTGCAGGTTACTTGTTATCCAAGCG-3′) and then inserted into SfiI/EcoRI-digested pGADT7 and NotI/PstI-digested pRK11-FLAG, respectively. To generate pGADT7-DNAJB6a, the coding sequence of DNAJB6 isoform a (DNAJB6a) was amplified by PCR using the primers DNAJB6a-F1 (5′-TGGCCATGGAGGCCATGGTGGATTACTATGAAGTTCTAG-3′) and DNAJB6a-R (5′-ATTGAATTCTGCAGGCTAGTGATTGCCTTTGGT-3′) and then cloned into SfiI/EcoRI-digested pGADT7. The coding sequence of DNAJB6a was also amplified by primers DNAJB6a-F2 (5′-AAGCGGCCGCGATGGTGGATTACTATGAAGTTCTAG-3′) and DNAJB6a-R and then inserted into NotI/PstI-digested pRK11-FLAG to generate pRK11-FLAG-DNAJB6a, which was used for expression in human cells. The identities of all constructs were confirmed by restriction digestion profile and sequencing. Constructs pGBKT7-UL70 and pCMV-Myc-UL70 that were used for the yeast two hybrid screen and for expression in human cells were generated as described previously [41].

### Cells, viruses, antibodies

Human glioblastoma U251 cells (formally known as ATCC U373MG) were purchased from American Type Culture Collection (ATCC) (Manassas, VA) (http://www.atcc.org/CulturesandProducts/CellBiology/MisidentifiedCellLines/tabid/683/Default.aspx.). U251 cells, foreskin fibroblasts (HFFs), HeLa cells, and 293T cells were cultured in Dulbecco's modified Eagle medium (DMEM) containing 10% newborn calf serum, 1% penicillin, and 1% phytomycin, as described previously [41]. HCMV (strain Towne and Towne_BAC_) was propagated in HFFs and U251 cells [43]. Mouse monoclonal anti-Myc and anti-FLAG antibodies were purchased from Sigma, Inc. (St. Louis, MO). Rabbit polyclonal anti-Myc antibody and the antibodies against DNAJB6a and b were purchased from Santa Cruz Biotech, Inc. (Santa Cruz, CA) and Genetex (San Antonio, TX). Alexa Fluor 488-, Alexa Fluor 555-, and Alexa Fluor-647-labeled secondary antibodies were purchased from Molecular Probes, Inc. (Portland, Oregon) and Invitrogen (Carlsbad, CA). The monoclonal antibodies that react with HCMV proteins UL44, IE1, and UL99 have been described previously [54].

To generate the anti-UL70 monoclonal antibody, the encoding sequence of UL70 was cloned into the NdeI/BamHI digested pET-28a(+) construct (Novogen, Madison, WI). The His-tagged UL70 protein was expressed in *E.coli* BL21 (DE3) strain, purified by gel electrophoresis, and then injected into BALB/C mouse. After immunizing BALB/C mouse three times, spleen cells were isolated and fused with myeloma cells. Positive fused cells were selected by HAT culture medium and ELISA screening. The resulting monoclonal antibody was collected from the supernatants of the culture medium of positive hybridoma cell cultures and kept in –20°C. The properties of the antibody to react with the UL70 protein were further characterized by Western blot analyses, co-IP, and indirect immunofluorescence assays.

To generate U251-6a, U251-6b, and U251-C cell lines, DNAs of constructs pCMV-DNAJB6a, pCMV-DNAJB6b, and the empty vector were co-transfected into U251 cells with DNA of LXSN vector [55]. At 48–72 hours postinfection, neomycin was added to the culture medium at a final concentration of 600 µg/ml. Cells were subsequently selected in the presence of neomycin for two weeks and neomycin-resistant cells were cloned [56], [57]. The levels of DNAJB6 in individual cell clones were determined by Western analysis.

### Yeast two-hybrid screen

*Saccharomyces cerevisiae* AH109, control vectors pGADT7, pGADT7-T and pGBKT7-p53, and a human fetal brain cDNA library were purchased from Clontech (Mountain View, CA). Plasmid pGBKT7-UL70 containing the GAL4 DNA-binding domain was transformed into AH109 containing a cDNA library derived from human fetal brain. Positive clones were selected on synthetic dropout (SD) medium lacking four nutrients tryptophan, leucine, adenine and histidine (SD-minus Trp/Leu/Ade/His, QDO) and tested for β-galactosidase activity by colony-lift filter assay according to the manufacturer's instructions and following the procedures as described previously [44]. DNA constructs that contained the sequence coding for the interaction partners of UL70 (designated as pACT2-cDNA) from positive colonies were extracted and the human gene sequences in the pACT2-cDNA constructs were determined with the sequencing primer (5′-AATACCACTACAATGGAT-3′) [41], [44].

### Coimmunoprecipitation (co-IP) and western blot analysis

Constructs were cotransfected using Lipofectamine (Invitrogen, Carlsbad, CA). At 48 hours posttransfection, cell lysates were harvested and co-IP experiments were performed using the ProFound Mammalian FLAG Tag IP/Co-IP and c-Myc Tag IP/Co-IP kits following the manufacturer's protocol (Pierce, Rockford, IL) [44]. To carry out Western analysis, the denatured polypeptides from cell lysates or co-IP were separated by sodium dodecyl sulfate-polyacrylamide gel electrophoresis (SDS-PAGE) and transferred electrically to nitrocellulose membranes [54], [55]. Membranes were blocked in 5% dried milk in PBS plus 0.2% Tween-20. Detection of tagged proteins was performed by incubation with different dilutions of primary antibodies. The proteins on the membranes were stained subsequently using a Western chemiluminescent substrate kit (GE Healthcare) and quantitated with a STORM840 PhosphorImager (GE Healthcare) or a Gel Documentation Station (BioRad, Hercules, CA) [54], [55]. A dilution series of the samples was analyzed and the results were compared in order to accurately determine the protein levels. The experiments were in duplicate and repeated three times. Quantitation was performed in the linear range of protein detection [54], [55].

### Transfection of siRNA into cells

Cells (n = 1×10^5^) were seeded in 12 well plates and transfected with chemically synthetic anti-DNAJB6a 6a-siRNA, anti-DNAJB6b 6b-siRNA, and control C-siRNA molecules (Ribobio Co. Ltd, Guangzhou, China). The 6a-siRNA molecules were a pool of three siRNAs that targeted different unique regions of the DNAJB6a mRNA while the 6b-siRNA molecules were a pool of two siRNAs that targeted different unique regions of the DNAJB6b mRNA (Table S2 in Text S1). For each well, 3 µl 20 mM siRNA and 3 µl Lipofectamine (Invitrogen) were diluted in 50 µl and 12 µl Opti-MEM (Invitrogen), respectively. After 5 minutes at room temperature, both solutions were combined. After 20 minutes, 400 µl prewarmed Opti-MEM was added to each transfection reaction mixture, which was subsequently added to the cells. At 10 hours posttransfection, siRNA-containing medium was removed. Cells were washed and incubated with complete DMEM supplemented with 10% fetal bovine serum for 14 hours, and the transfection was repeated once. Forty-eight hours after the second round of transfection, cells were either infected or prepared for analysis by immunoblotting.

### Immunofluorescence assays

Cells grown on glass coverslips were transfected with the different constructs using Lipofectamine (Invitrogen), and were then mock infected or infected with HCMV at a multiplicity of infection (MOI) of 0.5 to 1 at 24 hours posttransfection. After 72 hours posttransfection, cells were washed three times with washing buffer (0.1% bovine serum albumin (BSA) in PBS), fixed and permeabilized with PBS containing 4% paraformaldehyde and 0.2% Triton X-100 for 15–20 minutes at room temperature, and then washed three times with washing buffer followed by incubation with blocking buffer (5% BSA in PBS) for 1 hour at room temperature. Finally, the cells were incubated with appropriate primary antibodies (1∶100 dilution in washing buffer) overnight at 4°C. The cells were washed three times with washing buffer and incubated with secondary antibody Alexa Fluor 488 IgG, Alexa Fluor 555 IgG, or Alexa Fluor 647 IgG for 1 hour at room temperature. Nuclear staining was performed with 4′,6-diamidino-2-phenylindole (DAPI) (Invitrogen). Confocal images were collected using an Olympus confocal FV1000 microscope in separate channels and analyzed using the Olympus Fluoview software. In some of the experiments, images were captured with a Nikon Eclipse TE300 microscope using a SPOT RT Slider camera and imaging software (Diagnostic Instruments, Inc., Sterling Heights, MI) [41]. The digital images were subsequently merged with the SimplePCI software (Compix Inc., Sewickley, PA). Representative images are shown. Cells exhibiting different patterns of UL70 localization were counted manually and the percentages of the numbers of cells were determined from three independent experiments.

### Viral infection and assays for viral growth and gene expression

Cells (n = 1×10^6^) were either mock-infected or infected with HCMV (MOI = 0.5–5) in an inoculum of 1.5 ml of DMEM supplemented with 1% fetal bovine serum. The inoculum was replaced with DMEM supplemented with 10% fetal bovine serum after 2 hour incubation with cells. To determine the level of viral growth, the cells and medium were harvested at 5 days postinfection and viral stocks were prepared by adding an equal volume of 10% (vol/vol) skim milk, followed by sonication. The titers of the viral stocks were determined by infecting 1×10^5^ human foreskin fibroblasts and counting the number of plaques 10–14 days after infection. The values obtained were averages from triplicate experiments. To study viral gene expression, cells (*n* = 1×10^5^) were infected with HCMV at an MOI of 1. Protein extracts were prepared from the infected cells at 12–72 hrs postinfection, and analyzed by Western blot experiments as described previously [54].

### Cytoplasmic and nuclear extract preparation

Cells that were either mock-infected or infected with HCMV were harvested at different time points postinfection, and resuspended in buffer A (10 mM HEPES (pH 7.4), 10 mM KCl, 1 mM dithiothreitol, 0.6% NP-40) at 4°C for 10 minutes. After centrifugation at 1,000×g for 5 minutes, the supernatant was collected as the cytoplasmic fraction and the nuclear fraction was prepared by resuspending the remaining pellet in buffer B (20 mM HEPES (pH 7.4), 150 mM NaCl, 1 mM dithiothreitol). Equal amounts of cytoplasmic and nuclear extracts were used for immunoblotting of UL70, actin, and histone H1.

### Quantitative real-time PCR and HCMV DNA level analysis

Quantitative real-time PCR (qPCR) analysis of viral DNA was carried out as described elsewhere [58]. Briefly, DNA was isolated from mock-infected or HCMV-infected cells using a DNeasy tissue kit (Qiagen) according to the manufacture's instructions. The levels of intracellular viral DNAs were quantified with a primer pair [P53 (5′- GTCAGCGTTCGTGTTTCCCA-3′) and P33 (5′-GGGACACAACACCGTAAAGC-3′)] and a probe for amplifying the HCMV UL83 sequence. The probe (5′-[FAM]CCCGCAACCCGCAACCCTTCATG[TAMRA]-3′) was labeled at the 5′ end with a FAM and at the 3′ end with the fluorescent quencher TAMRA and purchased from Applied Biosystems Inc. (Foster City, CA). Amplification was performed in a 50-µl reaction mixture (which contained 21 µl of DNA extract, 25 µl of 2× *Taq*man Universal PCR Master Mix (Applied Biosystems Inc.), 1 µl of each primer at 10 µM, 2 µl of the fluorogenic probe at 5 µM) using either the iCycler Real-Time PCR Detection System (Bio-Rad, Hercules, CA) or an ABI 7500 device (Applied Biosystems Inc., Foster City, CA). Thermal cycling conditions were as follows: 50°C for 2 min, 95°C for 15 min, and 45 cycles of 95°C for 30 sec and 60°C for 1 min. The number of viral genomes was normalized to the number of cellular copies of β-actin with a previously described set of primers and probe [58]. Unknown sample values were determined on the basis of a standard curve of known copy numbers of UL83 (Towne_BAC_) and β-actin (pβ-actin). The PCR results were derived from three independent experiments.

## Supporting Information

Figure S1 **Coimmunoprecipitation of transiently expressed viral proteins and endogenous cellular DNAJB6a.** Human U251 cells were transfected with a plasmid expressing Myc-tagged viral proteins UL70 or UL25, and then infected with HCMV (MOI = 1) at 48 hours posttransfection. Cellular lysates were prepared at 48–72 hours postinfection. The input protein samples (50 µg) (Input) (lanes 1, 4, 7, and 10) and samples (10 µg) that were either immunoprecipitated with anti-Myc (IP (anti-Myc))(lanes 2, 5, 8, and 11) or anti-DNAJB6a antibodies (IP (anti-DNAJB6a))(lanes 3, 6, 9, and 12) were separated on SDS-containing polyacrylamide gels, and assayed with Western blot analysis using anti-Myc (anti-Myc)(A) and anti-DNAJB6a antibodies (anti-DNAJB6a)(B), respectively.
(TIF)

Figure S2 **Coimmunoprecipitation of native untagged viral proteins and cellular DNAJB6a during HCMV infection.** Human U251 cells were infected with HCMV (MOI = 1) and cellular lysates were prepared at 48–72 hours postinfection. The input protein samples (90 µg) (Input) (lanes 1, 4, 7, and 10) and samples (40 µg) that were either immunoprecipitated with anti-UL44 (IP (anti-UL44))(lanes 2 and 5), anti-UL70 (IP (anti-UL70))(lanes 8 and 11), or anti-DNAJB6a antibodies (IP (anti-DNAJB6a))(lanes 3, 6, 9, and 12) were separated on SDS-containing polyacrylamide gels, and assayed with Western blot analysis using anti-UL44 (anti-UL44)(lanes 1–3), anti-UL70 (anti-UL70)(lanes 7–9), and anti-DNAJB6a antibodies (anti-DNAJB6a)(lanes 4–6 and 10–12), respectively.
(TIF)

Figure S3 **Mapping of the domains of DNAJB6 that interact with UL70 by coimmunoprecipitation.** Human U251 cells were co-transfected with a combination of two plasmids expressing Myc-tagged UL70 protein and the full length or truncated FLAG-tagged DNAJB6a proteins, and then infected with HCMV (MOI = 1) at 48 hours posttransfection. Cellular lysates were prepared at 48–72 hours postinfection. The input protein samples (80 µg) (Input) (lanes 1, 4, and 7) and samples (15 µg) that were either immunoprecipitated with anti-Myc (IP (anti-Myc)) (lanes 3, 6, and 9) or anti-FLAG antibodies (IP (anti-FLAG)) (lanes 2, 5, and 8) were separated on SDS-containing polyacrylamide gels, and assayed with Western blot analysis using the anti-FLAG antibody (anti-FLAG).
(TIF)

Figure S4 **Co-localization of untagged UL70 and DNAJB6a and DNAJB6b expressed in human cells.** Cells were transfected with the construct pCMV-UL70 containing the sequence of UL70 alone (A) and in the presence of the constructs pCMV-DNAJB6a and pCMV-DNAJB6b containing the sequences of DNAJB6 (a and b) (B and C), fixed at 48 hours posttransfection, stained with anti-UL70, anti-DNAJB6a, and anti-DNAJB6b antibodies, and visualized using a microscope. The images of UL70 (b, e, i), DNAJB6a (f) and DNAJB6b (j), and the nuclei stained with DAPI (a, d, h) were used to generate the composite images (c, g, k). The images show different levels of magnification.
(TIF)

Figure S5 **Cellular localization of untagged UL44 and DNAJB6a and DNAJB6b expressed in human cells.** Cells were transfected with the construct pCMV-UL44 containing the sequence of UL44 alone (A) and in the presence of the constructs pCMV-DNAJB6a and pCMV-DNAJB6b containing the sequences of DNAJB6 (a and b) (B and C), fixed at 48 hours posttransfection, stained with anti-UL44, anti-DNAJB6a, and anti-DNAJB6b antibodies, and visualized using a microscope. The images of UL44 (b, e, i), DNAJB6a (f) and DNAJB6b (j), and the nuclei stained with DAPI (a, d, h) were used to generate the composite images (c, g, k). The images show different levels of magnification.
(TIF)

Figure S6 **Effect of the expression of DNAJB6 on the distribution of untagged UL70 in nuclear and cytoplasmic fractions.** Different cells (e.g. parental U251 cells, U251-6a and U251-6b cells, and 6a- and 6b-siRNA treated cells) were transfected with pCMV-UL70 in the absence and presence of siRNAs. At 48 hours posttransfection, cells were infected with HCMV (MOI = 1). At 48–72 hours postinfection, cells were harvested and separated into nuclear and cytoplasmic fractions. Equivalent amounts of each fraction were analyzed by immunoblotting with the anti-UL70 antibody. The purity of the nuclear and cytoplasmic fractions was assayed by immunoblotting with anti-histone H1 and anti-Actin, respectively. The membranes were reacted with antibodies and subsequently stained using a Western chemiluminescent substrate kit (GE Healthcare) and quantitated with a STORM840 PhosphorImager (GE Healthcare) or a Gel Documentation Station (BioRad, Hercules, CA) [54], [55]. A dilution series of the samples was analyzed and the results were compared in order to accurately determine the protein levels. Quantitation was performed in the linear range of protein detection [54], [55]. The experiments were in duplicate and repeated three times. The standard deviation is indicated by the error bar.
(TIF)

Text S1 **Supporting tables.** **Table S1.** The percentages of the numbers of cells in which UL70 was found to be localized in the nuclei (nuclei), cytoplasm (cytoplasm), or both (nuclei/cytoplasm). Cells were either transfected with pCMV-Myc-UL70 (Myc-UL70) or pCMV-UL70 (UL70), then infected with HCMV, stained with anti-Myc or anti-UL70 respectively, and visualized using a microscope. The experimental procedures were described in Materials and Methods. **Table S2.** The siRNA molecules used in the study. The 6a-siRNA molecules were a pool of three siRNAs (6a-1, 6a-2, and 6a-3) that targeted different regions of the DNAJB6a mRNA while the 6b-siRNA molecules were a pool of two siRNAs (6b-1 and 6b-2) that targeted different regions of the DNAJB6b mRNA. The oligonucleotides were designed and chemically synthesized by Ribobio Co. Ltd (Guangzhou, China). The positions of the target sequences by 6a-siRNA and 6b-siRNA molecules shown are at the unique regions of the DNAJB6a and DNAJB6b mRNAs, respectively [38].
(DOC)
