# Supplementary figures and images for: A Hsp40 Chaperone Protein Interacts with and Modulates the Cellular Distribution of the Primase Protein of Human Cytomegalovirus
Source: PLoS Pathog. 2012 Nov 1;8(11):e1002968. doi: 10.1371/journal.ppat.1002968 (PMC3486897; doi:10.1371/journal.ppat.1002968)

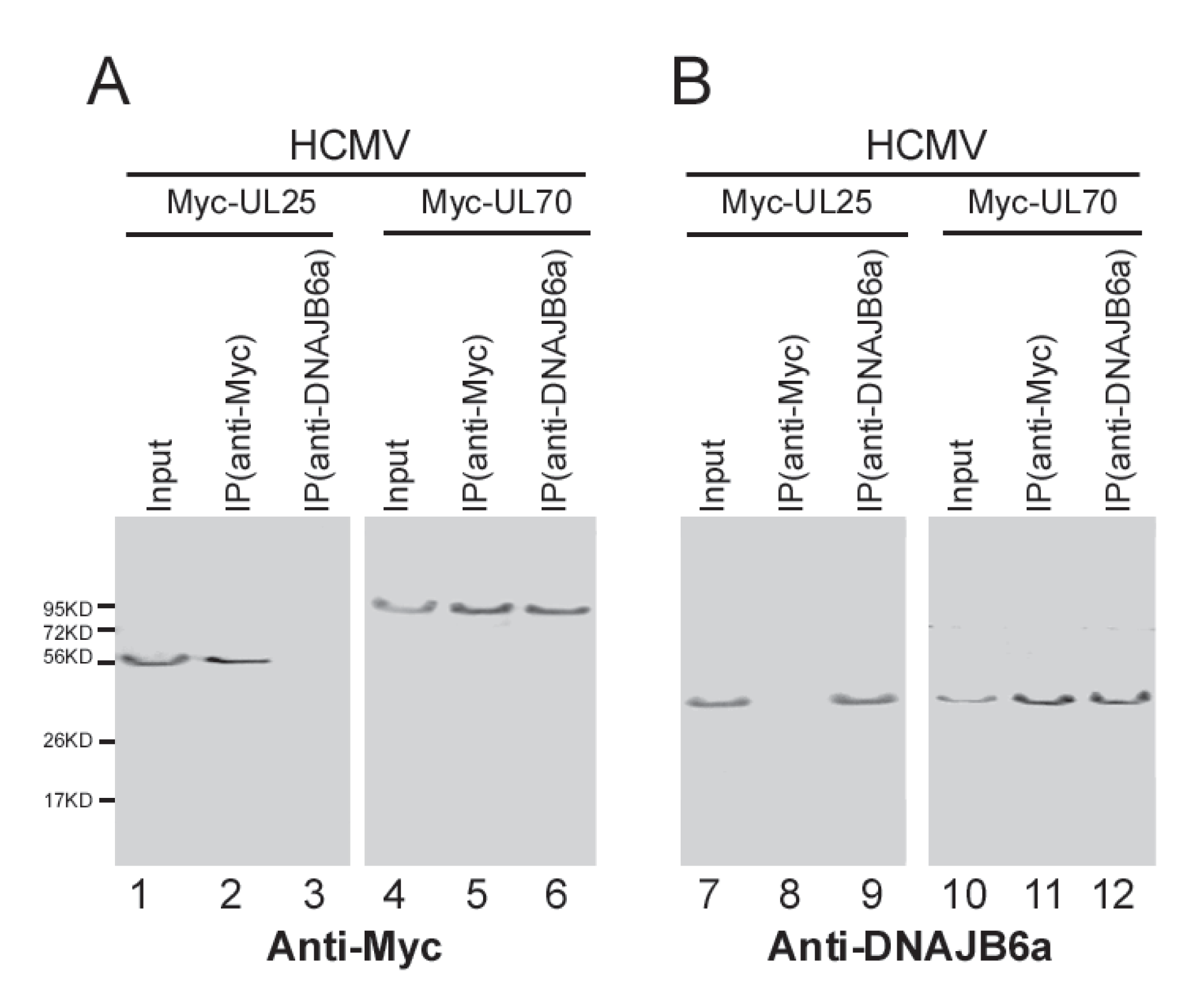

Supplement: Figure S1 — Coimmunoprecipitation of transiently expressed viral proteins and endogenous cellular DNAJB6a. Human U251 cells were transfected with a plasmid expressing Myc-tagged viral proteins UL70 or UL25, and then infected with HCMV (MOI = 1) at 48 hours posttransfection. Cellular lysates were prepared at 48–72 hours postinfection. The input protein samples (50 µg) (Input) (lanes 1, 4, 7, and 10) and samples (10 µg) that were either immunoprecipitated with anti-Myc (IP (anti-Myc))(lanes 2, 5, 8, and 11) or anti-DNAJB6a antibodies (IP (anti-DNAJB6a))(lanes 3, 6, 9, and 12) were separated on SDS-containing polyacrylamide gels, and assayed with Western blot analysis using anti-Myc (anti-Myc)(A) and anti-DNAJB6a antibodies (anti-DNAJB6a)(B), respectively. (TIF) [file ppat.1002968.s001.tif]

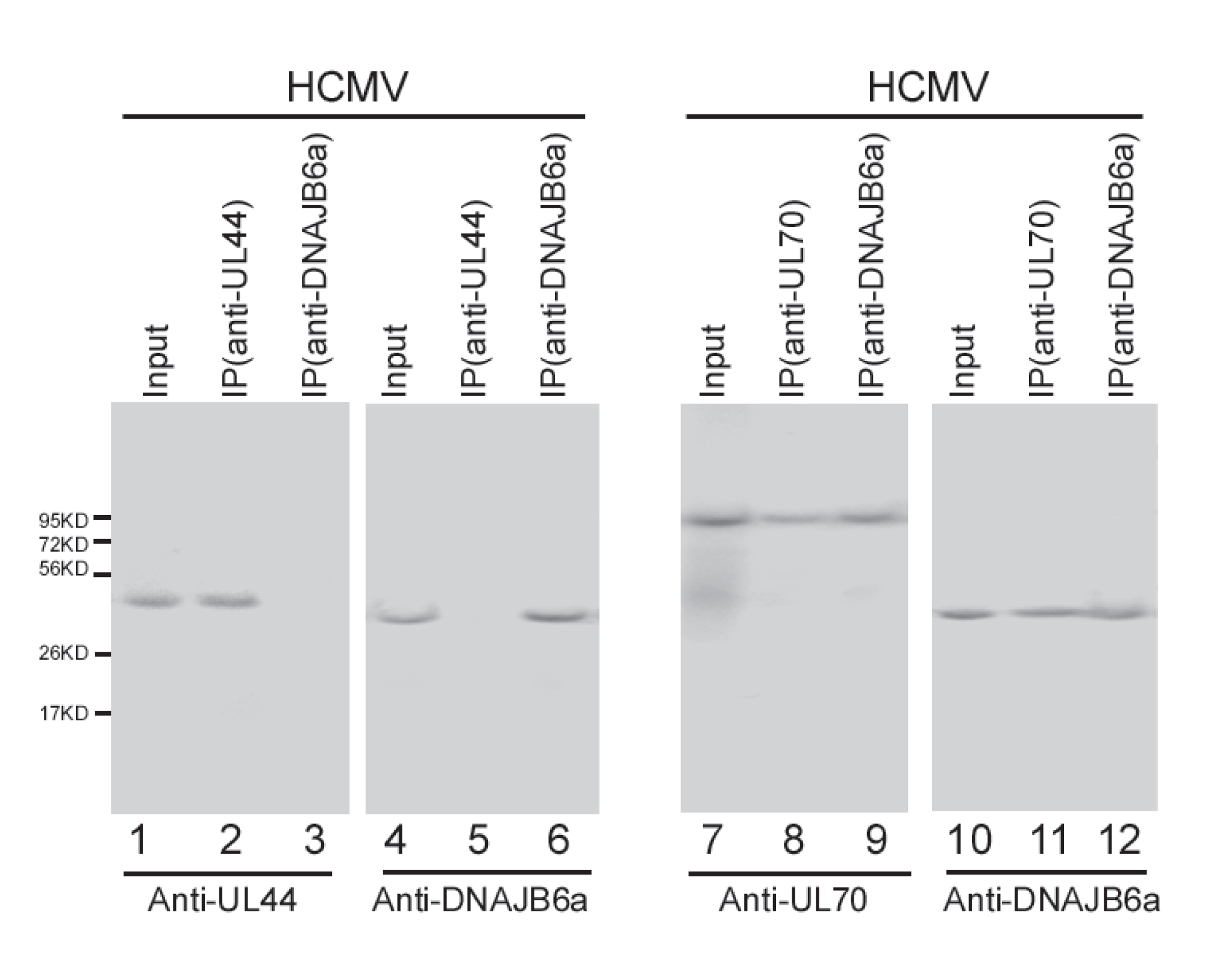

Supplement: Figure S2 — Coimmunoprecipitation of native untagged viral proteins and cellular DNAJB6a during HCMV infection. Human U251 cells were infected with HCMV (MOI = 1) and cellular lysates were prepared at 48–72 hours postinfection. The input protein samples (90 µg) (Input) (lanes 1, 4, 7, and 10) and samples (40 µg) that were either immunoprecipitated with anti-UL44 (IP (anti-UL44))(lanes 2 and 5), anti-UL70 (IP (anti-UL70))(lanes 8 and 11), or anti-DNAJB6a antibodies (IP (anti-DNAJB6a))(lanes 3, 6, 9, and 12) were separated on SDS-containing polyacrylamide gels, and assayed with Western blot analysis using anti-UL44 (anti-UL44)(lanes 1–3), anti-UL70 (anti-UL70)(lanes 7–9), and anti-DNAJB6a antibodies (anti-DNAJB6a)(lanes 4–6 and 10–12), respectively. (TIF) [file ppat.1002968.s002.tif]

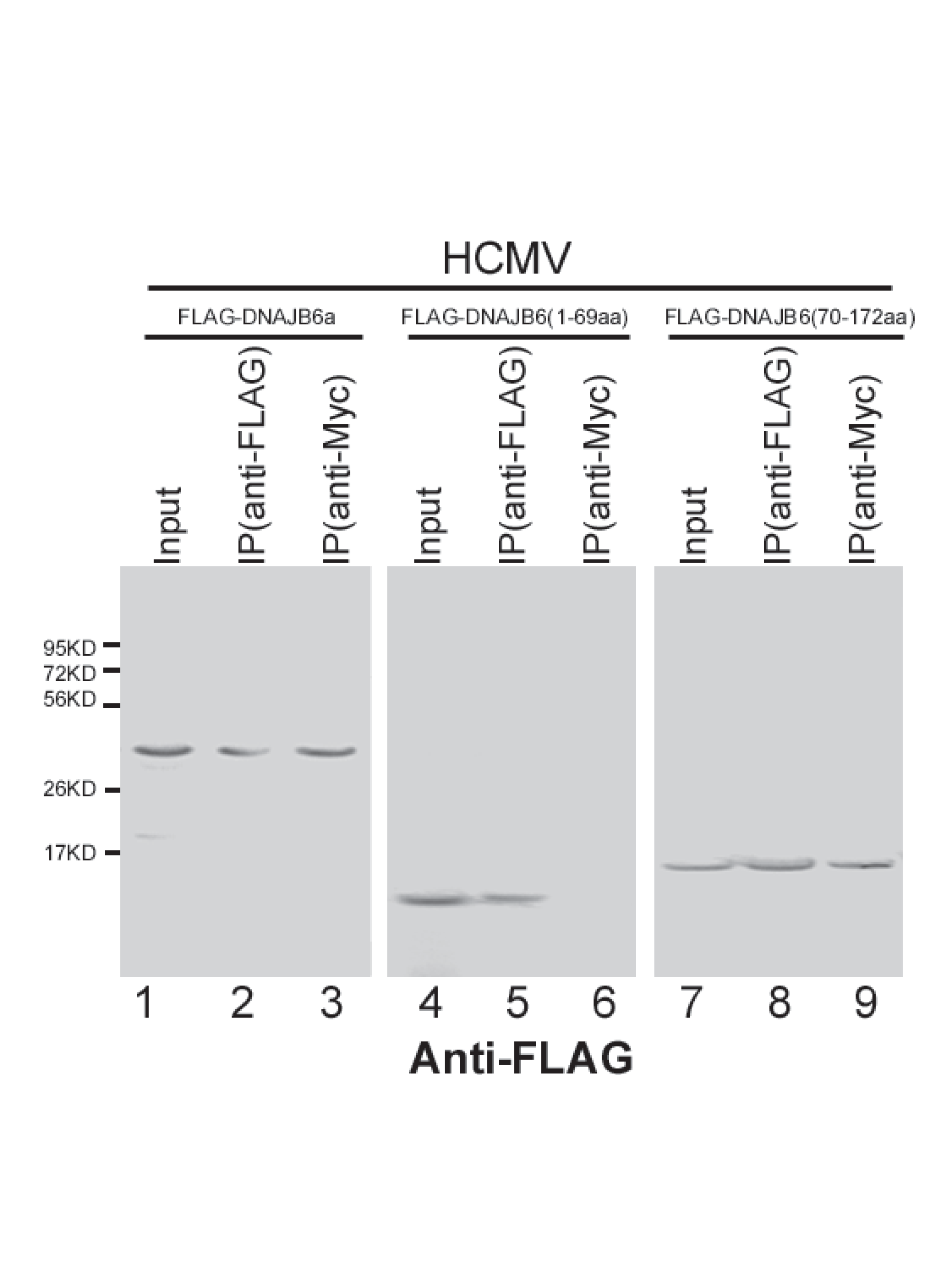

Supplement: Figure S3 — Mapping of the domains of DNAJB6 that interact with UL70 by coimmunoprecipitation. Human U251 cells were co-transfected with a combination of two plasmids expressing Myc-tagged UL70 protein and the full length or truncated FLAG-tagged DNAJB6a proteins, and then infected with HCMV (MOI = 1) at 48 hours posttransfection. Cellular lysates were prepared at 48–72 hours postinfection. The input protein samples (80 µg) (Input) (lanes 1, 4, and 7) and samples (15 µg) that were either immunoprecipitated with anti-Myc (IP (anti-Myc)) (lanes 3, 6, and 9) or anti-FLAG antibodies (IP (anti-FLAG)) (lanes 2, 5, and 8) were separated on SDS-containing polyacrylamide gels, and assayed with Western blot analysis using the anti-FLAG antibody (anti-FLAG). (TIF) [file ppat.1002968.s003.tif]

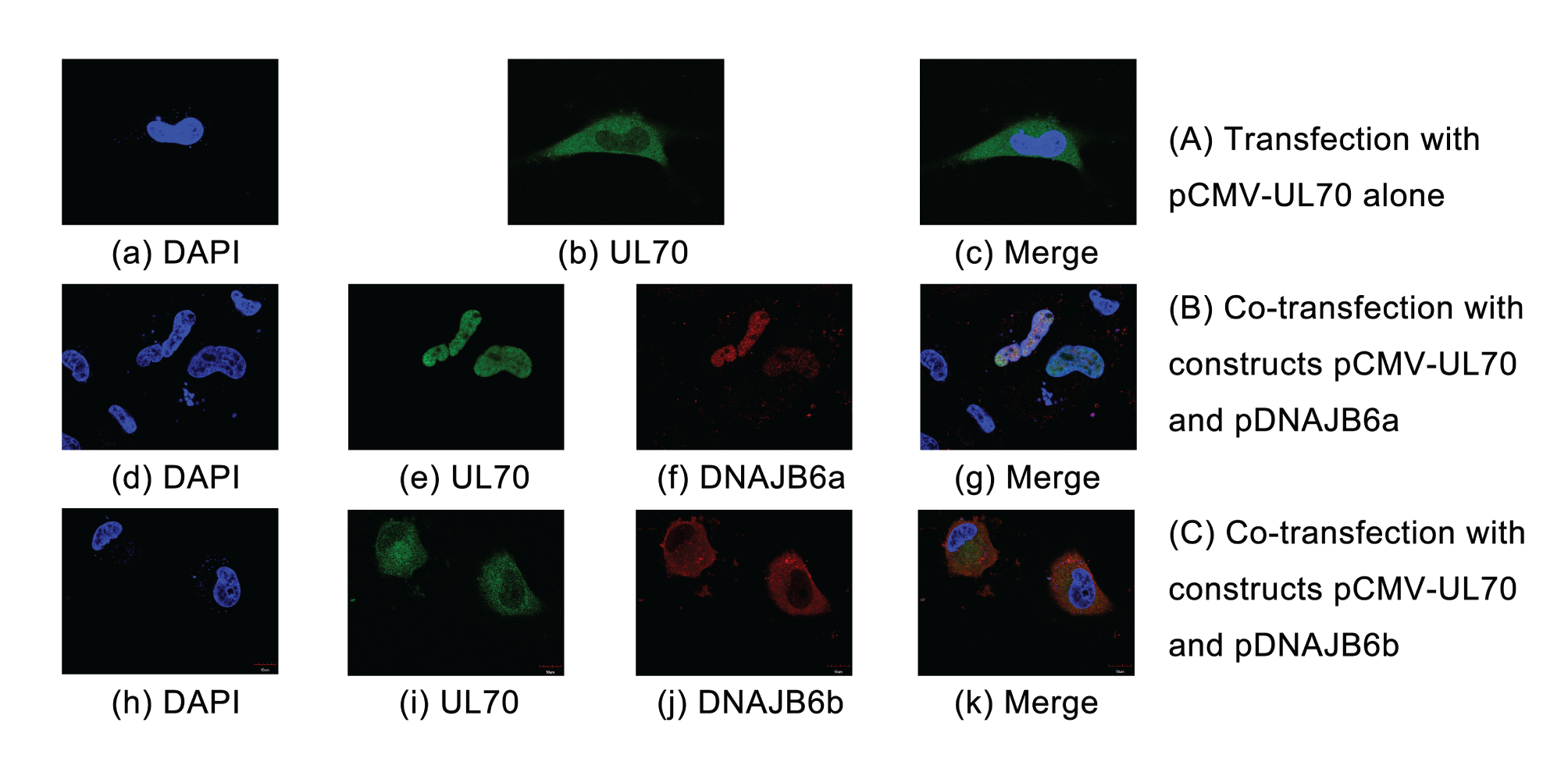

Supplement: Figure S4 — Co-localization of untagged UL70 and DNAJB6a and DNAJB6b expressed in human cells. Cells were transfected with the construct pCMV-UL70 containing the sequence of UL70 alone (A) and in the presence of the constructs pCMV-DNAJB6a and pCMV-DNAJB6b containing the sequences of DNAJB6 (a and b) (B and C), fixed at 48 hours posttransfection, stained with anti-UL70, anti-DNAJB6a, and anti-DNAJB6b antibodies, and visualized using a microscope. The images of UL70 (b, e, i), DNAJB6a (f) and DNAJB6b (j), and the nuclei stained with DAPI (a, d, h) were used to generate the composite images (c, g, k). The images show different levels of magnification. (TIF) [file ppat.1002968.s004.tif]

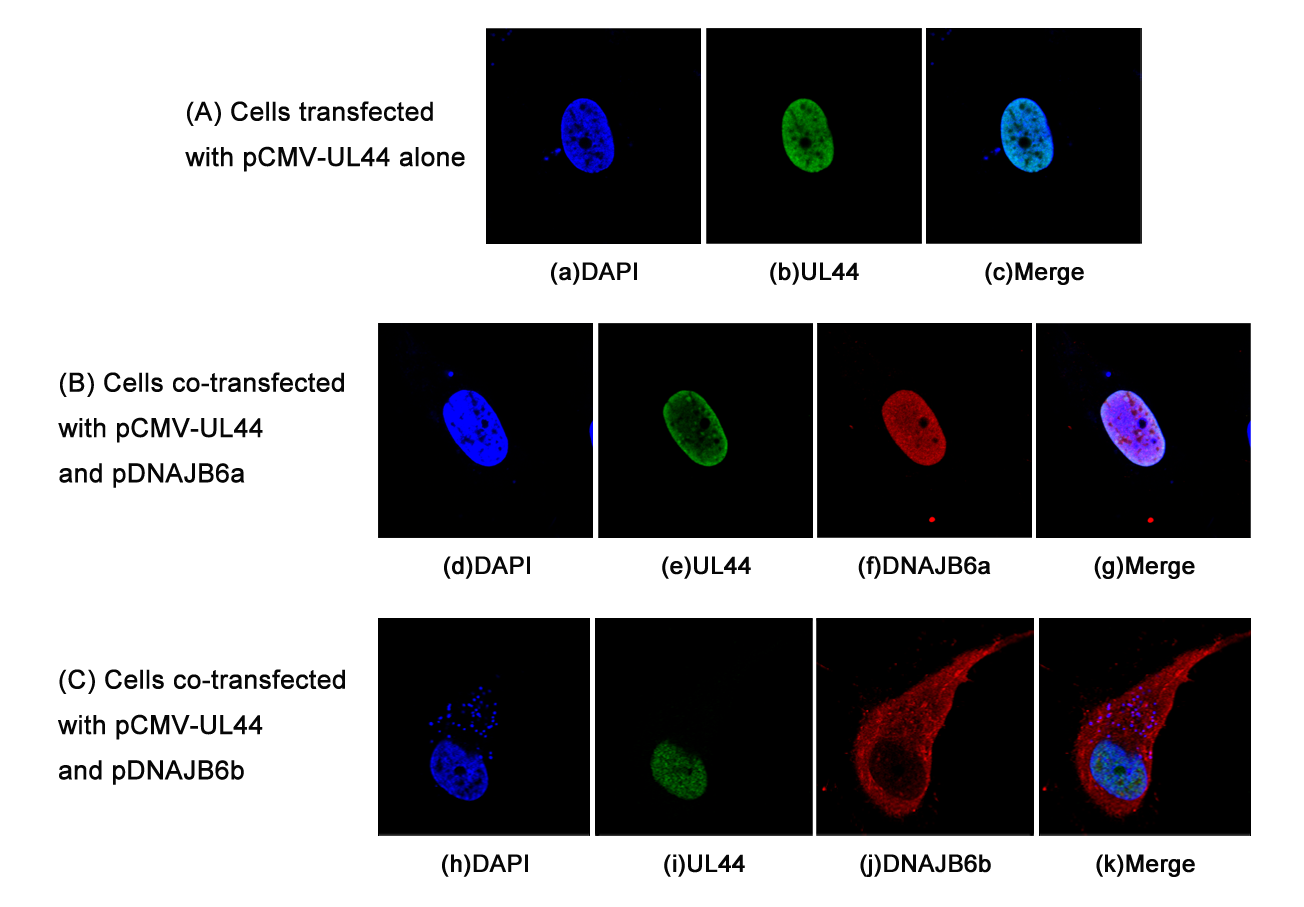

Supplement: Figure S5 — Cellular localization of untagged UL44 and DNAJB6a and DNAJB6b expressed in human cells. Cells were transfected with the construct pCMV-UL44 containing the sequence of UL44 alone (A) and in the presence of the constructs pCMV-DNAJB6a and pCMV-DNAJB6b containing the sequences of DNAJB6 (a and b) (B and C), fixed at 48 hours posttransfection, stained with anti-UL44, anti-DNAJB6a, and anti-DNAJB6b antibodies, and visualized using a microscope. The images of UL44 (b, e, i), DNAJB6a (f) and DNAJB6b (j), and the nuclei stained with DAPI (a, d, h) were used to generate the composite images (c, g, k). The images show different levels of magnification. (TIF) [file ppat.1002968.s005.tif]

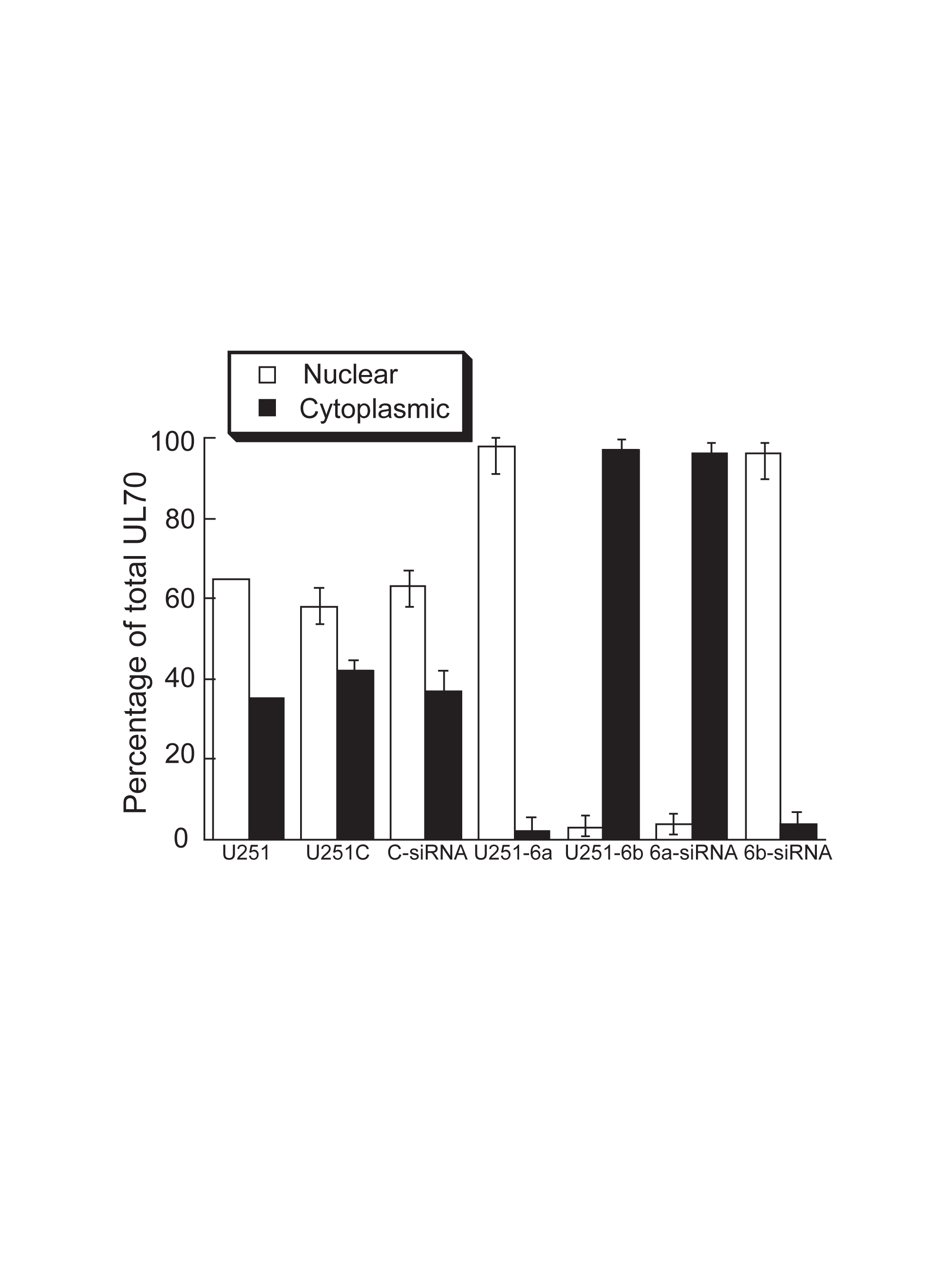

Supplement: Figure S6 — Effect of the expression of DNAJB6 on the distribution of untagged UL70 in nuclear and cytoplasmic fractions. Different cells (e.g. parental U251 cells, U251-6a and U251-6b cells, and 6a- and 6b-siRNA treated cells) were transfected with pCMV-UL70 in the absence and presence of siRNAs. At 48 hours posttransfection, cells were infected with HCMV (MOI = 1). At 48–72 hours postinfection, cells were harvested and separated into nuclear and cytoplasmic fractions. Equivalent amounts of each fraction were analyzed by immunoblotting with the anti-UL70 antibody. The purity of the nuclear and cytoplasmic fractions was assayed by immunoblotting with anti-histone H1 and anti-Actin, respectively. The membranes were reacted with antibodies and subsequently stained using a Western chemiluminescent substrate kit (GE Healthcare) and quantitated with a STORM840 PhosphorImager (GE Healthcare) or a Gel Documentation Station (BioRad, Hercules, CA) [54], [55]. A dilution series of the samples was analyzed and the results were compared in order to accurately determine the protein levels. Quantitation was performed in the linear range of protein detection [54], [55]. The experiments were in duplicate and repeated three times. The standard deviation is indicated by the error bar. (TIF) [file ppat.1002968.s006.tif]
